# Safety, Pharmacokinetics, Translational and Molecular Mechanistic Insights on the Prostate Cancer Recurrence Suppressor Pseurotin A

**DOI:** 10.3390/molecules30193963

**Published:** 2025-10-02

**Authors:** Oliver C. McGehee, Hassan Y. Ebrahim, Sharon Meyer, Nehal A. Ahmed, Chandra Mohan Reddy Muthumula, Dalal Dawud, Judy A. King, Amal Kaddoumi, Khalid A. El Sayed

**Affiliations:** 1 Department of Basic Pharmaceutical and Toxicological Sciences, College of Pharmacy, University of Louisiana at Monroe, 1800 Bienville Drive, Monroe, LA 71201, USA; mcgeheoc@warhawks.ulm.edu (O.C.M.); hebrahim@vcom.edu (H.Y.E.); atefkhaledahmedabdn@warhawks.ulm.edu (N.A.A.); mcmohanreddy8@gmail.com (C.M.R.M.); dawuddr@warhawks.ulm.edu (D.D.); 2 Department of Biomedical Sciences, Discipline of Pharmacology, Edward Via College of Osteopathic Medicine, Monroe, LA 71203, USA; 3 DeBusk College of Osteopathic Medicine, Lincoln Memorial University, 9737 Cogdill Road, Knoxville, TN 37932, USA; judy.king@lmunet.edu; 4 Department of Pharmacology and Toxicology, Medical College of Georgia, Augusta University, 1201 Goss Lane, Augusta, GA 30912, USA; akaddoumi@augusta.edu

**Keywords:** differentially expressed genes, PCSK9, prostate cancer, pseurotin A, recurrence, RNA-sequencing, ubiquitin-proteasome system

## Abstract

Elevated cholesterol levels play important mitogenic roles. Pseurotin A (PsA) is a fermentation product that has recently been reported as a dual inhibitor of proprotein convertase subtilisin/kexin type 9 (PCSK9) secretion and protein-protein interaction (PPI) with the LDLR. PsA showed a high acute safety profile and therapeutic potential against metastatic castration-resistant prostate cancer (mCRPC). The study aims to uncover the chronic safety, distribution, and anti-mCRPC genomic and molecular mechanistic insights of PsA. A 90-day chronic safety assessment of PsA up to 80 mg/kg in *Swiss albino* mice showed no signs of hematological, biochemical, or major organ toxicity. PsA demonstrated rapid intravenous distribution and elimination in *Swiss albino* mice. PsA is biodistributed to multiple key organs but was not detected in the brain, indicating its inability to cross the blood-brain barrier. PsA effectively suppressed the recurrence of nude mice xenografted mCRPC, which was subjected to a neoadjuvant docetaxel and enzalutamide regimen, followed by surgical excision. Collected PsA and vehicle control-treated recurrent tumors were subjected to RNA-sequencing and pathway enrichment analysis (PEA) of differentially expressed genes (DEGs). PsA-treated tumors revealed multiple significantly enriched pathways associated with promoting tumor apoptosis and inhibiting both invasion and migration. The PPI network analyses for the downregulated DEGs displayed prominent networks of genes associated with the ubiquitin-proteasome system. Results provide comprehensive mechanistic and preclinical validations for PsA’s potential as a novel PC recurrence suppressive lead entity.

## 1. Introduction

Prostate cancer (PC) is the second most prominent type of cancer in men, which accounts for >35,000 annual deaths [1]. PC survival and growth are driven by androgen receptor (AR) signaling. Thus, PC treatment initially revolves around the depletion of circulating androgens, usually through castration. However, PC cells can adapt to androgen deprivation therapy (ADT), which may result in progression to the more aggressive castration-resistant prostate cancer (CRPC). Evidence for the role of cholesterol in PC pathogenesis has been progressively growing, with several studies identifying hypercholesterolemia as a potentially modifiable risk factor in PC development [2]. Cholesterol is the biosynthetic precursor to androgens and a key substrate for de novo steroidogenesis in prostate cells. The mCRPC cells rely on de novo cholesterol biosynthesis rather than transcellular uptake, and recent studies have shown that androgen-independent (AI) PC cells’ growth can be promoted by the low-density lipoprotein cholesterol (LDL-C) availability [3].

The proprotein convertase subtilisin/kexin type 9 (PCSK9) regulates LDL-C levels by binding to the low-density lipoprotein receptor (LDLR), resulting in its degradation by the lysosome. The LDLR in the liver represents the primary pathway for the removal of plasma LDL-C [4]. Thus, an overexpression of PCSK9 in this organ can lead to higher levels of circulating LDL-C. PCSK9 inhibitors not only reduce systemic levels of cholesterol but also reduce the supply of cholesterol to cancer cells and are therefore indicated for familial hypercholesterolemia in which statins are not effective [4].

Alirocumab and evolocumab are currently FDA-approved, marketed humanized monoclonal antibodies (mAbs) that interfere with the PCSK9-LDLR PPI, considerably lowering LDL-C levels [4]. However, mAbs are large molecules that are unable to cross the plasma membrane and fail to act intracellularly. Furthermore, manufacturing of mAbs is expensive and should be formulated in parenteral forms. Therefore, there are several non-mAb PCSK9 inhibitors being developed as potential alternative treatments for reducing circulating LDL-C.

**Scheme 1:**
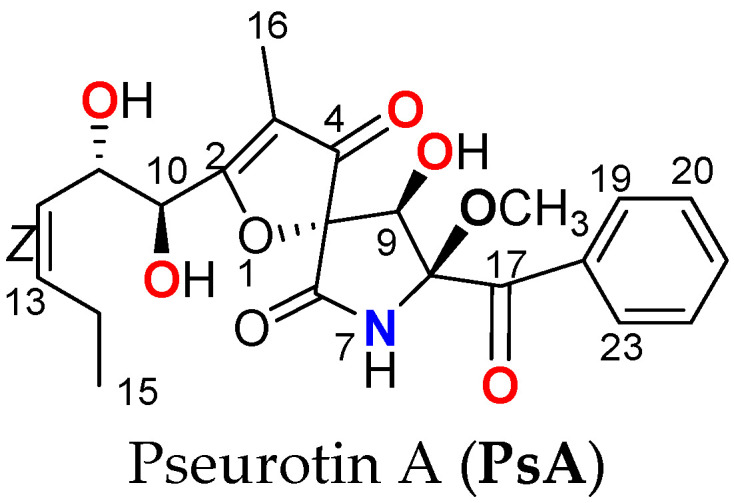
Chemical structure of PsA.

Pseurotins are spiroheterocyclic γ-lactam alkaloids isolated from *Aspergillus fumigatus* and other fungi fermentation. Among the topmost bioactive pseurotins are pseurotin A (PsA, Scheme 1) and the related pseurotin D. Both PsA and pseurotin D showed relevant antitumor and immunomodulatory effects [5]. PsA dually inhibited PSCK9 expression and interaction with LDLR in mouse livers and various tumor models with a promising safety profile [6,7,8,9]. PsA showed in vitro dose-dependent reduction of PCSK9 expression in the hormone-dependent BT-474 luminal B breast cancer (BC) cells [6]. PsA suppressed the in vivo progression and recurrences of the BT-474 BC cells in a nude mouse xenograft model by reducing the liver and plasma PCSK9 levels and normalizing the liver LDLR level [6]. This established PsA as a novel first-in-class small molecule lead dually reducing the PCSK9 expression and suppressing its PPI with LDLR in hormone-dependent BC and possibly other hormone-dependent malignancies [6]. Subsequent studies revealed PsA’s suppression of PCSK9 expression, migration, and colony formation in CRPC cell lines PC-3 and 22Rv1 [7,8]. PsA lowered the progression, locoregional and distant recurrences of the CRPC cell lines PC-3 and CWR-R1ca in vivo in xenografted nude mouse models, validating the novel concept of targeting the PCSK9-LDLR axis to control CRPC pathogenesis [7,8]. Studies on the PsA in vitro toxicity against RWPE-1 and CCD 841 CoN human non-tumorigenic prostate and colon cells proved cellular death reached at the 10-fold level of literature-reported therapeutic activity [9]. PsA-mediated induction of non-malignant cells’ apoptosis was at very high concentrations [9]. The Up-and-Down acute toxicity procedure showed a PsA LD_50_ value of >550 mg/kg in male and female *Swiss albino* mice [9]. A 14-day acute toxicity assay of PsA 10, 250, and 500 mg/kg by oral gavage versus vehicle control in *Swiss albino* mice reported neither major organ toxicity in the liver, lung, heart, brain, or kidneys nor any alarming changes in the hematological and biochemical parameters [9]. The 500 mg/kg female dosing group showed a 45% decrease in body weight after 14 days but displayed no other signs of toxicity [9]. Single oral doses of PsA, up to 50-fold the therapeutic dose, did not pose acute organ toxicity in *Swiss albino* mice [9]. While this study displayed this wide PsA acute safety index, there is still a dire need to assess its chronic safety since the effect of PCSK9 inhibitors on neurodegenerative diseases is still controversial [10].

The hydroxymethylglutaryl-CoA (HMG-CoA) reductase inhibitory-LDLR expression promoter statins are standard interventions for hypercholesterolemia [11]. Literature linked HMG-CoA reductase inhibition with lowered cognitive performance and potential long-term harmful neurocognitive effects [11]. Many patients using statins encounter muscle-related rhabdomyolysis, leading to intolerance and treatment discontinuation [11,12]. PC-specific mortality risk was lower among statin users; however, statin use did not reduce the risk of PC recurrence [12]. Statins also proved to stimulate PCSK9 transcription, which adversely affects their long-term efficacy [12].

PCSK9 binds to the LDLR, resulting in its degradation by the lysosomes, yet there are conflicting views on whether the ubiquitin-proteasome system (UPS) could also be involved in PCSK9-induced LDLR degradation. The sterol-responsive nuclear liver X receptor (LXR) inhibits the LDLR pathway through induction of the E3 ubiquitin ligase, known as the inducible degrader of the LDLR (Idol), which triggers the ubiquitination of the LDLR and ensures its degradation [13]. LDLR ubiquitination was detected following PCSK9-targeting treatments, suggesting that a different E3 ligase other than Idol is involved in PCSK9-induced LDLR ubiquitination [14]. LDLR degradation mediated by PCSK9 does not require proteasome function or ubiquitination of the LDLR cytoplasmic tail [15]. These inconsistent findings suggest that the clear mechanism of PCSK9-mediated LDLR degradation is yet to be delineated.

The UPS serves as the most prominent driver for the regulation of proteolysis in all eukaryotic cells. The UPS plays a key role in regulating a wide range of cellular pathways that include apoptosis, neuronal signaling, growth, and proliferation through the cell cycle and immune response [16]. Dysregulation of UPS-linked pathways correlated with neurodegenerative disorders like Alzheimer’s disease, Parkinson’s, and certain cancers [17]. Clinical trials on the proteasome inhibitor bortezomib have demonstrated significant improvement in survival rates in patients suffering from multiple myeloma [18]. This established proteasome inhibition as a key molecular target in cancer therapy, which was further validated by subsequent studies in a variety of cancers [19,20,21,22]. A dysfunctional UPS and associated genes are prominent molecular markers in mCRPC cells [21,23,24].

The uniqueness of PsA as the first small-molecule dual suppressor of PCSK9 expression and inhibitor of its PPI with LDLR highlights its future potential as a novel cancer recurrence suppressive lead entity. This study presents the PsA comprehensive chronic toxicity, bioavailability, biodistribution, recurrence suppressive efficacy in a clinical-mimicking mCRPC model, and RNA-sequencing-based mechanistic molecular insights.

## 2. Results

### 2.1. Effects of PsA Treatment on Swiss Albino Mice Body Weight

Mice were placed on a high-fat diet (HFD, 11% total fat content) over the study duration. Following 90-day daily oral dosing of PsA, the body weight of the male and female treatment groups did not show any significant changes over the sixteen time points measured versus the vehicle control (Supplementary Figures S1 and S2). The female 10 mg/kg group showed a slight drop in body weight from day 77 to 83, but saw a reversal back to the mean before sacrifice on day 90 of dosing. The body weight showed minor fluctuations from week to week (<10%) while slightly increasing in all four groups over the 90 days of dosing (Supplementary Figure S2). The loss in body weight is widely accepted as a potential early empirical marker for organ toxicity [9]. Thus, this indicates a lack of major organ toxicity resulting from daily oral doses of PsA not exceeding 80 mg/kg.

### 2.2. Effects of PsA Treatments on Swiss Albino Mice Behavior

Daily oral doses of PsA over 90 days had no observable effects on mice’s behavioral responses (grooming, alertness, vocalization, anti-social), neuromuscular responses (body posture, gait, righting reflex, startle response, tonic/clonic convulsions), nor autonomic responses (respiratory rate, piloerection, cyanosis, diarrhea) at any of the time points observed [25]. A senior toxicologist with >20 years of animal testing was present within the first month after dosing and consulted for any changes in mouse responses. Mice were observed closely for 1 h after oral dosing every day until day 30. After day 30, the mice were observed closely for 30 min after each oral dosing until termination of the experiment on day 90. In mammalian safety studies, the first observable indicators of potential toxicity are changes in behavioral, autonomic, or neurological responses. In many safety studies, this portion of the assessment is often lacking in defined and implemented systems for documenting indicators of distress [26]. Therefore, we utilized the mouse grimace scale in order to more accurately quantify pain and discomfort across the 90 days of repeated dosing [26]. The mice’s behavior remained consistently healthy and showed no signs of change over the 90-day dosing duration. The mice displayed neither neurological nor autonomic response abnormalities, and displayed no signs of grimace or discomfort in their facial expressions.

### 2.3. Evaluation of Relative Animal Organ Weight

Following a 90-day course of daily oral administration of PsA, the vital organs such as the liver, kidneys, spleen, lungs, heart, and brain were collected after sacrifice and weighed. The kidneys and lungs were both weighed together. The results showed a significant decrease in average heart weight for the male 10 mg/kg and 40 mg/kg treatment groups compared to the vehicle control group (Supplementary Table S1). This was the only significant change in relative organ weights observed in the male dosing groups as compared to the vehicle control (Supplementary Table S1). There were no significant changes in the relative organ weights for any of the six organs measured in the female mice treatment groups as compared to vehicle control (Supplementary Table S2, Dunnett’s test, *p* < 0.05).

### 2.4. Analysis of Mice Hematological and Biochemical Parameters After PsA 90-Day Daily Oral Dosing

**Figure 1:**
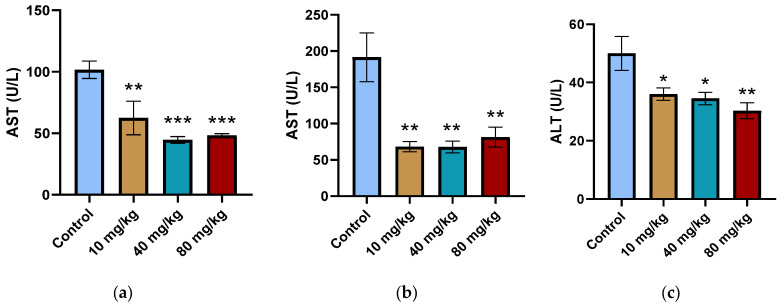
Effects of PsA treatments on mice’s liver enzymes. (**a**) Plasma AST levels (mean ± SEM) of male *Swiss albino* mice treated with PsA. (**b**) Plasma AST levels (mean ± SEM) of female *Swiss albino* mice treated with PsA. (**c**) Plasma ALT levels (mean ± SEM) of female *Swiss albino* mice treated with PsA. Samples were collected in lithium heparin blood collection tubes and immediately centrifuged at 13,000× *g* for 10 min. Plasma was transferred to an Eppendorf tube free of lithium heparin and analyzed using the Beckman AU680 clinical chemistry analyzer system. Results for the biochemical parameters were analyzed separately by One-way Analysis of Variance (ANOVA) followed by multiple comparisons with Dunnett’s test. * Indicates statistical significance at *p* < 0.05, ** indicates statistical significance at *p* < 0.01, and *** indicates statistical significance at *p* < 0.001.

The aspartate aminotransferase (AST) and alanine aminotransferase (ALT) are prominent biochemical markers for detecting potential hepatotoxicity. AST level of all male dosing groups was significantly lower than that of the control (Figure 1a). This significant reduction of AST was mirrored in the treatment groups of the female mice (Figure 1b) but was accompanied by significantly lower plasma levels of ALT in all three treatment groups (Figure 1c). The treated males did not show a significant change in ALT levels as compared to the control (Supplementary Table S3, Supplementary Figure S3). The average AST levels for the male and female control mice were 150.8 ± 43.5 U/L and 197.3 ± 33.6 U/L, respectively (Supplementary Tables S3 and S4). The average ALT levels for the male and female control mice were 40.8 ± 4.0 U/L and 48.3 ± 5.8 U/L, respectively (Supplementary Tables S3 and S4).

**Figure 2:**
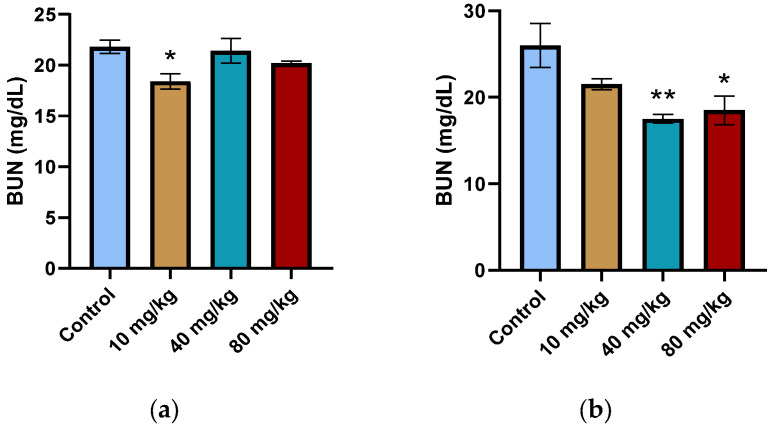
Effects of PsA treatments on mice’s blood urea nitrogen (BUN) levels. (**a**) Plasma BUN levels (mean ± SEM) of male *Swiss albino* mice treated with PsA. (**b**) Plasma BUN levels (mean ± SEM) of female *Swiss albino* mice treated with PsA. Samples were collected in lithium heparin blood collection tubes and immediately centrifuged at 13,000× *g* for 10 min. Plasma was transferred to an Eppendorf tube free of lithium heparin and analyzed using the Beckman AU680 clinical chemistry analyzer system. Results for the biochemical parameters were analyzed separately by One-way Analysis of Variance (ANOVA) followed by multiple comparisons with Dunnett’s test. * Indicates statistical significance at *p* < 0.05, and ** indicates statistical significance at *p* < 0.01.

The BUN levels of the male 10 mg/kg, female 40 mg/kg, and female 80 mg/kg dosing groups were significantly lower than those of control mice (Figure 2). The alkaline phosphatase (ALP) and plasma glucose levels of both the male and female treatment groups showed no significant changes over the 90 dosing days (Supplementary Tables S3 and S4).

### 2.5. Analysis of Mice Hematological Parameters After PsA 90-Day Daily Oral Dose

The effects of up to 80 mg/kg PsA on the hematological parameters of male mice and up to 10 mg/kg female mice following 90 days of daily oral dosing were studied (Supplementary Tables S5 and S6). The white blood cell count (WBC-H), red blood cell count (RBC-H), hemoglobin count (HGB), hematocrit (HCT), mean corpuscular hemoglobin concentration (MCHC), and platelet count (PLT) did not show any significant changes between the treatment and control groups (Supplementary Tables S5 and S6). The mean corpuscular volume (MCV) in the male 40 mg/kg treatment group was significantly higher than that of the control (Supplementary Figure S4).

### 2.6. Effects of PsA Treatments on Serum Cholesterol Levels in Swiss Albino Mice

**Figure 3:**
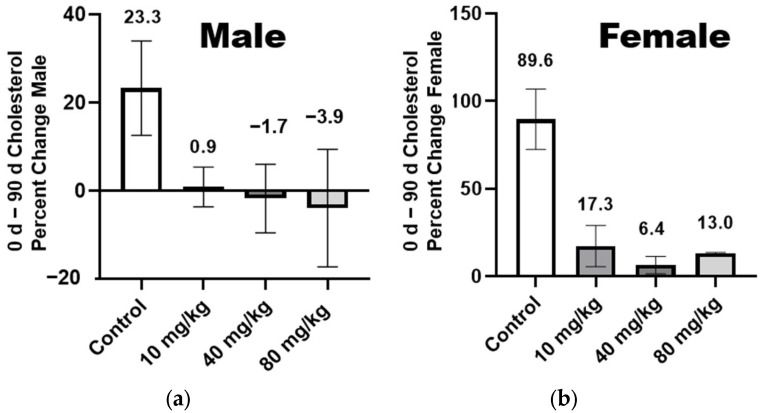
Comparative long-term anti-hypercholesterolemic effects of PsA treatments on HFD-fed *Swiss albino* mice. (**a**) Percent change of cholesterol levels in male *Swiss albino* mice after 90-day oral dosing of PsA. (**b**) Percent change of cholesterol levels in female *Swiss albino* mice after 90-day oral dosing of PsA. Average percent change for male and female groups plotted as mean ± SEM (*n* = 5).

The effects of 90-day daily oral administration of PsA on the plasma cholesterol levels of *Swiss albino* mice maintained on HFD are illustrated in Figure 3. The HFD effects were more pronounced in female control mice, displaying an 89.6% increase in cholesterol levels after 90 days, compared to only a 23.3% increase in male control mice. Daily dosing of PsA reduced the HFD-induced hypercholesterolemia in female mice by −80.7% in the 10 mg/kg group, −92.9% in the 40 mg/kg group, and −85.5% in the 80 mg/kg group relative to the control. The treated male mice plasma cholesterol levels displayed a greater relative reduction response versus the control group after the 90-day daily oral dosing, illustrating a decrease of −96.1% in the 10 mg/kg, −107.3% in the 40 mg/kg, and −116.7% in the 80 mg/kg groups.

### 2.7. Effects of Chronic Oral Dosing of PsA on Organ Histology

The liver, lungs, kidneys, heart, and brain were excised from the control and treatment groups after 90-day dosing and subjected to histological examination by a light microscope. Sections were analyzed for any signs of gross toxicity, including signs of necrosis, apoptosis, tissue lesions, and other organ-specific abnormalities. The results for all five organs analyzed up to the dose of 80 mg/kg showed no structural or morphological differences when compared to the vehicle control mice (Supplementary Figure S5). Greater emphasis was placed on observing the liver as the mice were subjected to HFD, as well as the liver being the organ with the highest expression of PCSK9 in the body [4,5,6,9]. Histopathological examination of the mouse livers revealed no signs of lipid droplet formation and was comparable with that of a normal liver in wild-type mice fed on a regular chow diet [27]. This was consistent across the control and the three treatment groups, with no signs of PsA treatment protecting or exacerbating the progression of steatosis. The mouse cerebral cortex, cerebellum, and hippocampus were evaluated under H&E staining, with special attention given to the hippocampus as it is a primary site for neurotoxicity. Evaluation of the hemi-coronal section of the hippocampus through the widest part of the brain can help ensure any CNS alterations will not be overlooked [28]. The pyramidal neuronal layer of each mouse hippocampus was uniformly arranged and structurally intact (Supplementary Figure S5e). Healthy glial cells were present in the molecular layer, with the nucleus of the neurons presenting no signs of disfiguration.

### 2.8. Optimization of a PsA Analytical HPLC Method with High Specificity for in Vivo Analysis

**Figure 4:**
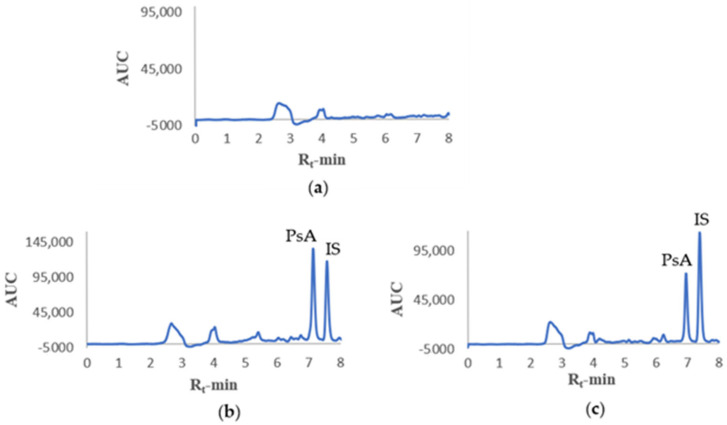
Optimization of quantitative HPLC analytical method for PsA in mice plasma. The *x*-axis represents the HPLC peak area under the curve measured at 254 nm. The *y*-axis represents peaks eluted between 0 and 8 min retention time. Chromatographic representation of (**a**) blank mouse plasma (**b**) blank mouse plasma spiked with 50 µg/mL PsA (R_t_ = 6.9 min) + internal standard (IS) (50 µg/mL, R_t_ = 7.4 min) (**c**) IV bolus 50 mg/kg PsA with IS extracted from mouse plasma sample collected at 5 min following the dosing.

Blank mouse plasma, blank mouse plasma spiked with PsA and internal standard (IS), in addition to a plasma sample withdrawn from a mouse at 5 min following IV administration of PsA, were analyzed to assess the specificity of the HPLC method (Figure 4). 2-Benzofuran carboxylic acid was selected as the IS based on its chemical stability, reproducibility, and optimal retention time. The retention time of PsA was 6.9 min, and the IS was 7.4 min, respectively. A good chromatographic resolution of mouse plasma extracts was achieved with no endogenous substances or metabolites interfering with the separation or quantitation of the desired analytes (Figure 4). The PsA extraction recovery was greater than 90% at the low, medium, and high concentrations (Table 1). The level of variability decreased as the concentration increased; however, this variability change was still in accordance with the guidelines for Bioanalytical Method Validation of the Food and Drug Administration [29]. The EtOAc-isopropanol (7:3) liquid-liquid extraction method proved effective in extracting PsA from mouse plasma samples.

### 2.9. Assessment of PsA Chemical Stability in Mice Plasma

PsA showed a high chemical stability profile in mouse plasma after three freeze-thaw cycles with minimal variations between runs (Table 2). PsA also displayed a good chemical stability profile after two weeks of storage in mouse plasma at −20 °C, yet with greater variation between samples run. PsA did not show adequate stability in mouse plasma at room temperature for 24 h, with over 50% concentration loss (Table 2). To account for this, all samples were extracted as quickly as possible and were stored at −20 °C if immediate extraction was not possible.

### 2.10. Calibration Curve and Lower Limits of Quantification

The peak area ratio of PsA to IS in mouse plasma was linear at 4–50 µg/mL PsA concentration range, with the regression equation y = 0.0232x + 0.1445 (r^2^ = 0.9932). The limit of detection (LOD) for PsA was found to be 0.5 µg/mL (S/N ≥ 3). The lower limit of quantification (LLOQ) was found to be 4 µg/mL (S/N ≥ 5).

### 2.11. PsA Analysis Accuracy and Precision

Intra-day and inter-day accuracy and precision for the validated method are displayed in Table 3. The intra-day coefficient of variation (% CV) for PsA showed a range from 1.96% to 3.36% and accuracy from 84.1% to 96.4%. The inter-day coefficient of variation for PsA ranged from 2.72% to 8.19% with an accuracy from 82.7% to 99.7%. Though the intra-day showed a higher precision, these results denote that the study is accurate, reliable, and reproducible.

### 2.12. Pharmacokinetics Study of PsA in Swiss Albino Mice Following Intravenous Administration

**Figure 5:**
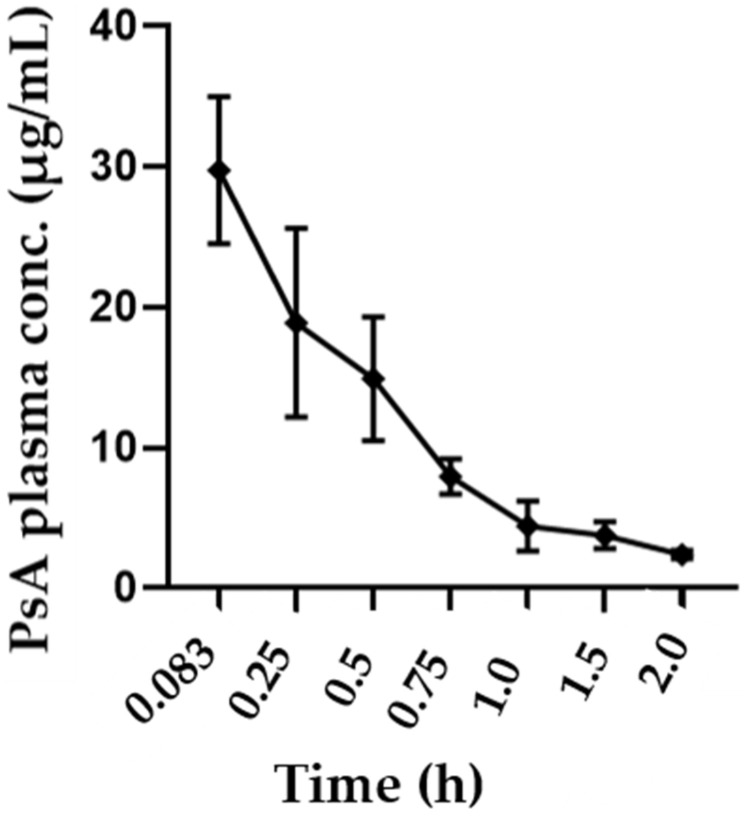
Mean plasma concentration of PsA after a single IV dose (50 mg/kg) administered in 24 healthy *Swiss albino* mice following 8 h fasting. PsA plasma drug concentration (µg/mL) displayed as mean ± SD across time points 0.083, 0.25, 0.5, 0.75, 1, 1.5, and 2 h (*n* = 7).

The pharmacokinetics (PKs) profile of PsA in mouse plasma following IV administration of PsA was successfully elucidated using the developed and validated analytical method. After recto-orbital injection of PsA at a dose of 50 mg/kg to mice, the mean plasma concentration—time profile was plotted (Figure 5). Relevant PK parameters were calculated (Table 4). The plasma concentration of PsA showed a rapid decline within the first 5 min, with all PsA traces undetectable by the HPLC method after 2 h. The average volume of distribution was 1.83 L/kg. The mean area for the plasma concentration—time curve from zero to last measurable plasma concentration (AUC_0–t_) was 19.06 µg h/mL, and the mean area under the curve—time curve from zero to infinity (AUC_t–∞_) was 20.9 µg h/mL.

### 2.13. Qualitative Tissue Distribution Study of PsA in Swiss Albino Mice

**Figure 6:**
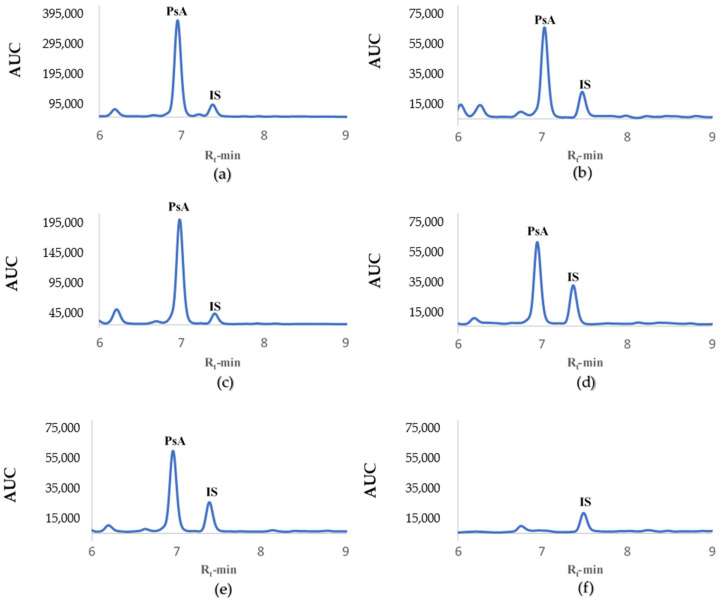
Chromatographic representation of plasma and multiple organs biodistribution of PsA in *Swiss albino* mice after IP dosing at 125 mg/kg versus internal standard (IS). The *x*-axis represents the HPLC peak area under the curve measured at 254 nm. The *y*-axis represents peaks eluted between 6 and 9 min retention time (R_t_). Representative chromatograms include *Swiss albino* mice (**a**) plasma, (**b**) liver, (**c**) kidney, (**d**) heart, (**e**) spleen, (**f**) brain.

The goal of this experiment was to assess the PsA organs-tissues distribution in *Swiss albino* mice and whether PsA would cross the blood-brain barrier (BBB) to reach the brain using the validated analytical HPLC method. Seven min following the IP dosing of PsA 125 mg/kg, multiple *Swiss albino* mice were sacrificed, organs were excised, weighed, homogenized, and subjected to the validated extraction and HPLC analytical methods. The time point of 7 min was chosen to account for the rapid distribution and elimination of PsA in the mouse body observed in the PK results. Chromatograms of the mouse plasma, liver, kidney, heart, spleen, and brain were examined for PsA peaks at R_t_ of 6.9 min (Figure 6). The collected and extracted mouse plasma showed a prominent PsA peak at 6.9 min followed by the IS peak at ~7.4 min (Figure 6a). Liver, kidney, heart, and spleen tissue samples each displayed a prominent PsA peak at ~6.9 min followed by the IS peak at ~7.4 min (Figure 6b–e). However, the brain tissue samples did not display PsA peak, indicating the inability of PsA to cross the BBB (Figure 6f). The detection of PsA in the liver, kidney, heart, and spleen confirmed its successful distribution into these organs. This experiment was not quantitative, and the failure of PsA to cross the BBB was tentatively consistent with its molecular weight > 400 Daltons, per Lipinski’s rule. This result, along with other data, helped to exclude PsA’s potential for CNS toxicity.

### 2.14. PsA Effectively Suppressed mCRPC Recurrences After Docetaxel and Enzalutamide Neoadjuvant Regimen Followed by Primary Tumors Surgical Excision

PsA was reported earlier by this study team to suppress the progression and recurrences of the mCRPCs PC-3 and CWR-R1ca cells in nude mice [7,8]. Neoadjuvant chemotherapy with the taxane docetaxel (DTX) followed by the second-generation androgen pathway inhibitor (API) ENZ are standard mCRPC intervention regimens. A clinically-mimicking model in male nude mice xenografted the mCRPC CWR-R1ca-Luc cells was used to test the PsA recurrence suppressive efficacy after the completion of a neoadjuvant 3-week subeffective regimen of DTX (once weekly, 10 mg/kg, IV) followed by 4-week daily oral use of the API ENZ (10 mg/kg). The primary tumors were surgically resected. Animals then received daily oral PsA at 10 mg/kg and 20 mg/kg versus vehicle control (VC) for the subsequent 60 days. Both PsA treatment groups showed significant suppression for the CWR-R1ca-Luc mCRPC locoregional recurrence, unlike the VC group (Table 5). Treated mice showed only one locoregional recurrence in each of the 10 and 20 mg/kg treatment groups, *n* = 5 and *n* = 7, respectively, unlike the VC group, which showed locoregional recurrence in 6 out of 7 group mice. Similarly, both PsA treatment groups showed effective suppression of the distant recurrences in the organs, especially in the bones, lung, and liver (Table 5). These specific organs are the favorite CRPC metastatic sites [7,8]. The used subeffective doses of DTX and ENZ were intended to expose the mCRPC cells to these standard interventions to ensure incomplete remission and mimic the human clinical situations in which the treatment fails, leading to tumor recurrence in several patients. Worth noting the wide range of VC group distant recurrences to bones and organs, validating the model’s success because distant recurrences are likely due to escaping the resistant tumor cells-tumor stem cells from the tumor microenvironment at the xenografting site through the host animal systemic circulation to distant organ tissues. Effective primary tumor surgical excision was confirmed by immediate post-surgery live animal imaging, and no leftover bioluminescent tumor tissues remained at the surgery site [11]. Study mice were not castrated because the CWR-R1ca cells are castration-resistant but hormone-sensitive. Leaving endogenous androgens enhanced the tumor aggression. Collected locoregionally recurred tumors in PsA 20 mg/kg and VC-treated groups were subjected to RNA-Seq bioinformatic analyses. The recurrent tumors in this model are phenotypically unique because their parent primary tumors had subeffective exposure to DTX and ENZ. Recurrence tumors normally have a more aggressive and resistant phenotypic profile. Collected recurrence tumors had a long-term exposure to PsA treatments and therefore reflected valid molecular effects.

### 2.15. Identification of PsA-Treatment-Induced Upregulated and Downregulated Genes

**Figure 7:**
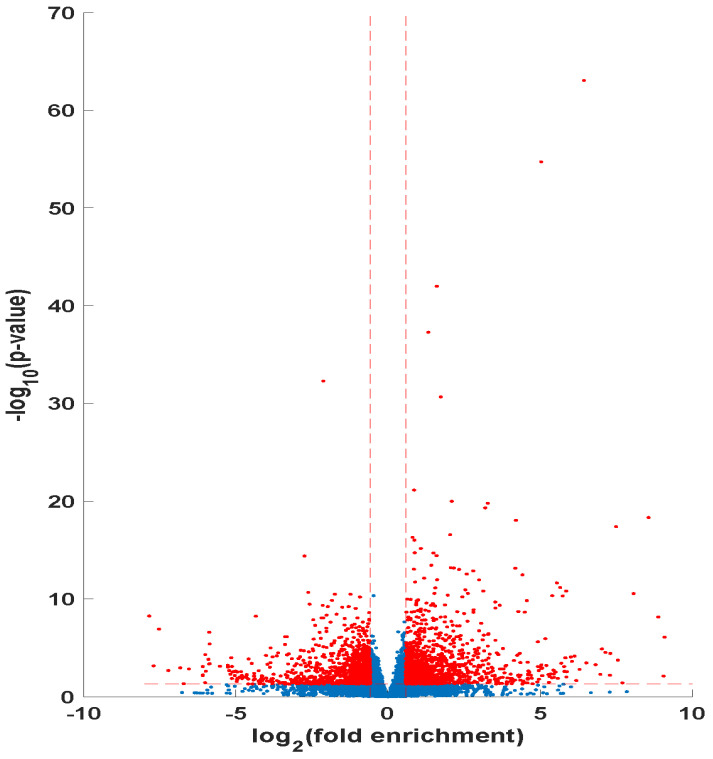
Volcano plot of the DEGs comparing the PsA versus VC treatments on the recurrent CWR-R1ca tumor tissues. Significantly upregulated and downregulated DEGs are colored red, while the non-significantly upregulated and downregulated DEGs are colored blue. The red vertical lines denote log fold-changes of −1.5 and 1.5, while the red horizontal line represents a negative log adjusted *p*-value of 0.05.

A cutoff criterion of log2FC ≥ 1.5 and log2FC ≤ −1.5, adjusted *p*-value ≤ 0.05, and FDR ≤ 0.1 was implemented following DESeq2 screening of the differentially expressed genes comparing between PsA-treated and VC-treated recurrent tumors. This resulted in a total of 838 differential genes being obtained. The PsA treatment significantly upregulated 486 genes and downregulated 352 genes. These genes are represented as dots in the volcano plot (Figure 7). The *x*-axis displays the positive and negative log2FC values, while the *y*-axis corresponds to the mean expression of the negative log10 adjusted *p*-value. The vertical hyphenated red line represents the 0.05 *p*-value cutoff, while the horizontal red hyphenated lines represent the log2FC ≥ 1.5 and log2FC ≤ −1.5. The dots in red are genes that met the cutoff criteria.

### 2.16. Gene Ontology Enrichment Analysis of Differentially Expressed Genes

**Figure 8:**
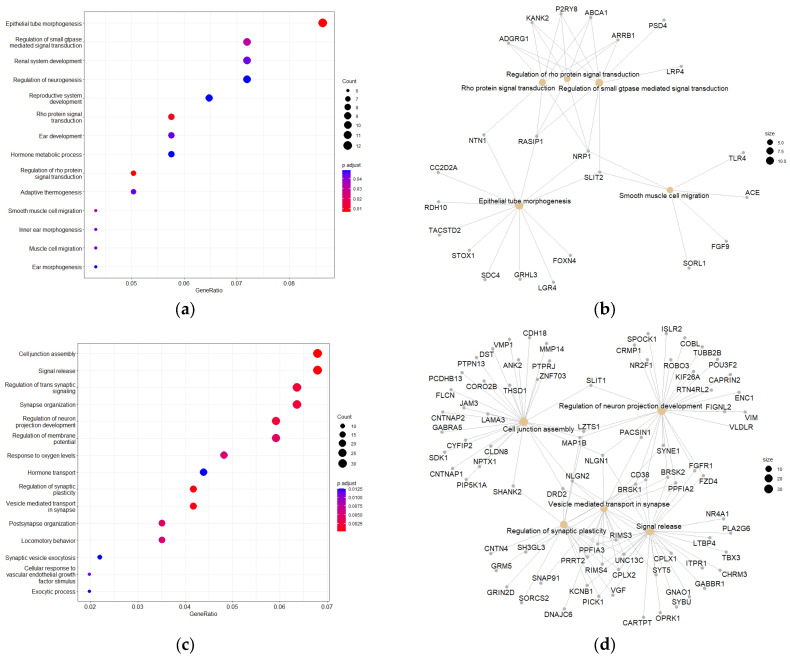
Gene Ontology Enrichment Analysis of DEGs in response to PsA treatment compared to the VC in the recurred mCRPC CWR-R1ca tumors. The *x*-axis for dot plots represents the ratio of significantly enriched DEGs associated with each biological process pathway. The size of the dot is proportional to the number of DEGs associated with each biological pathway (minimum gene count > 5) and colored from red to blue based on *p*-adjusted significance of each association (*p*-adj ≤ 0.05). (**a**) Dot plot of downregulated significantly enriched DEGs. (**b**) Cnet plot of downregulated significantly enriched DEGs associated with the top 5 most significantly enriched biological processes. (**c**) Dot plot of upregulated significantly enriched DEGs. (**d**) Cnet plot of upregulated significantly enriched DEGs associated with the top 5 most significantly enriched biological processes.

A Gene Ontology (GO) database consisting of 838,968 genes was utilized to identify biological processes, cellular components, and molecular functions that were significantly enriched within the DEGs. Separate GO enrichment analyses utilizing clusterProfiler and enrichR were performed for the upregulated and downregulated genes. A *p*-adjusted cutoff of <0.05 and a gene count cutoff of >5 were used to filter out the non-significant pathways. The *p*-value was adjusted based on the Benjamini–Hochberg procedure to reduce the number of false positives. About 23 out of 352 downregulated DEGs assigned to 14 GO pathways were enriched and displayed in Figure 8a (*p*-adj < 0.001). These genes (Figure 8b) were linked to the 5 most significant pathways (*p*-adj < 0.0001). Nearly 57 out of 486 upregulated DEGs assigned to 44 GO pathways were enriched. The 15 topmost significantly enriched pathways are displayed in Figure 8c (*p*-adj < 0.0001). The genes displayed in Figure 8d are linked to the 5 topmost significant pathways (*p*-adj < 0.00001). All significantly enriched genes that met the cutoff criteria for both the upregulated and downregulated DEGs were related to biological processes. The most significantly downregulated DEGs were associated with epithelial tube morphogenesis, Rho protein signal transduction, and regulation of Rho protein signal transduction (Figure 8a). The most significantly upregulated DEGs were involved in cell junction assembly, signal release, regulation of trans-synaptic signaling, synapse organization, and regulation of neuron projection development (Figure 8c).

### 2.17. Kyoto Encyclopedia of Genes and Genomes Pathway Enrichment Analysis

**Figure 9:**
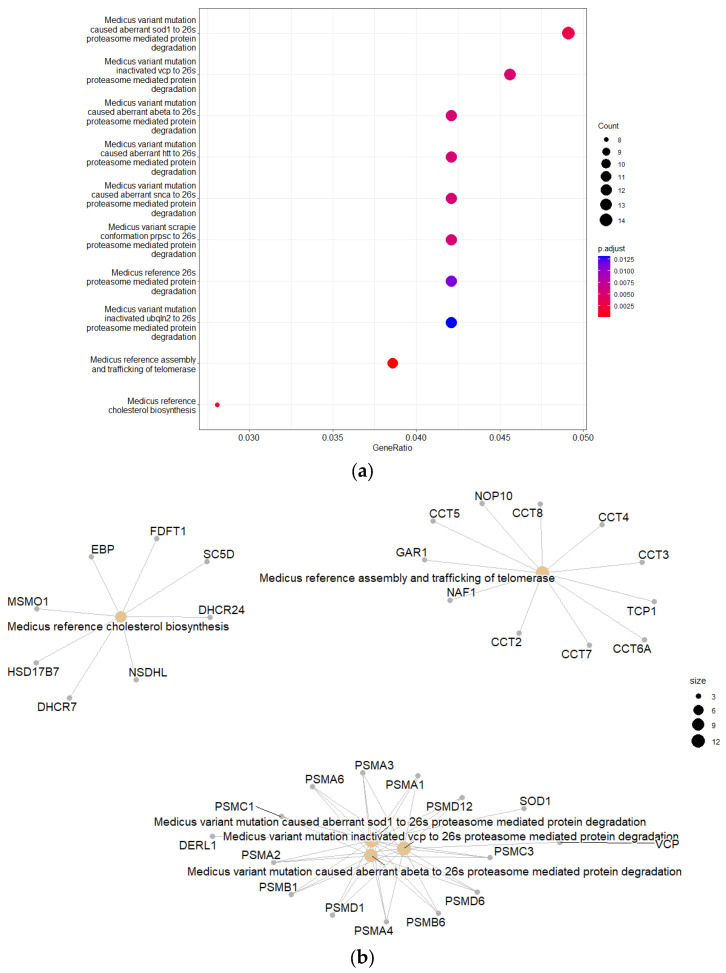
KEGG enrichment analysis of DEGs between PsA versus VC treatments in the mCRPC collected recurrent tumors. (**a**) Dot plot of downregulated significantly enriched DEGs. The *x*-axis represents the ratio of significantly enriched DEGs associated with each biological process pathway. The size of the dot is proportional to the number of DEGs associated with each biological pathway (minimum gene count > 5) and colored from red to blue based on *p*-adjusted significance of each association (*p*-adj ≤ 0.05). (**b**) Cnet plot of downregulated significantly enriched DEGs associated with the topmost 5 significantly enriched canonical pathways.

A KEGG medicus database consisting of 9662 genes was utilized to identify canonical pathways that were significantly enriched within the DEGs. Pathway enrichment analysis (PEA) for upregulated and downregulated DEGs was selected based on a log2FC ≥ 0.5 or ≤−0.5, adjusted *p*-value ≤ 0.05, and an FDR ≤ 0.1. This resulted in 2799 DEGs with 1435 upregulated and 1364 downregulated mapped to 658 pathways in the KEGG medicus database. Separate GO enrichment analyses utilizing clusterProfiler and enrichR were performed for the upregulated and downregulated genes. An adjusted *p*-value cutoff of <0.05 and gene count cutoff of >5 were used to filter out non-significant pathways. The *p*-value was adjusted based on the Benjamini–Hochberg procedure to reduce the number of false positives. 34 out of 1364 downregulated DEGs assigned to 10 KEGG pathways were significantly enriched (Figure 9a, *p*-adj < 0.01). These genes (Figure 9b) were linked to the 5 most significant pathways (*p*-adj < 0.005). No pathway enrichment was observed in the upregulated DEGs. The most significantly mapped pathway terms for the downregulated DEGs involved aberrant sod1 to 26S proteasome-mediated protein degradation, assembly and trafficking of telomerase, and cholesterol biosynthesis (Figure 9).

### 2.18. Protein-Protein Interaction and Gene Regulatory Functional Network Analysis

**Figure 10:**
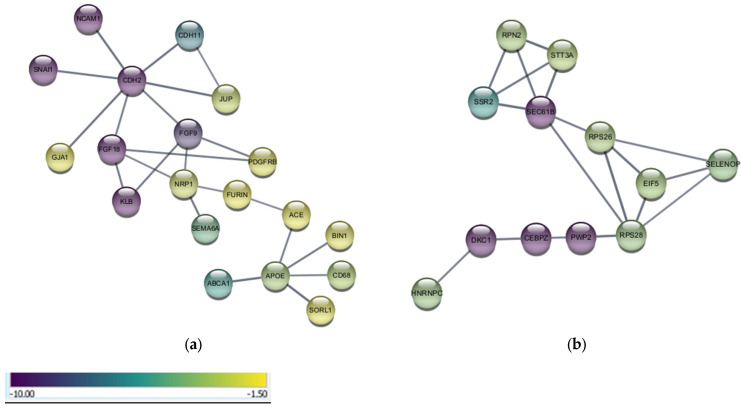
STRING functional network analysis of the downregulated DEGs in response to comparative PsA-VC treatments in the collected recurrent CWR-R1ca tumors. The cutoff criteria for the DEGs were log2FC ≥ 1.5 and log2FC ≤ −1.5, adjusted *p*-value ≤ 0.05, and FDR ≤ 0.1. The cutoff criteria for the STRING functional network analysis were 0.8. (**a**) The topmost prominent network displays 19 nodes and 22 edges. (**b**) The second most prominent network, representing 12 nodes and 18 edges. Circular nodes are labeled by gene and colored using a continuous mapping color gradient based on Log2FC ranging from −1.5 to −10 or lower.

The STRINGapp in Cytoscape was used to identify significant networks and interactions between DEGs comparing PsA versus VC-treated CWR-R1ca tumors. The cutoff criteria for the DEGs were log2FC ≥ 1.5 and log2FC ≤ −1.5, adjusted *p*-value ≤ 0.05, and FDR ≤ 0.1. This resulted in 342 nodes with 106 edges for the downregulated genes and 481 nodes and 151 edges for the upregulated genes (confidence cutoff > 0.8). There were 25 network-based visualizations for the downregulated DEGs, with 7 of the networks having at least 5 nodes and edges. The most prominent network had 19 nodes and 22 edges (Figure 10a), with the second most prominent network having 12 nodes and 18 edges (Figure 10b). About 32 network-based visualizations, with 4 of the networks having at least 5 nodes and edges observed for the upregulated genes. The topmost prominent network had 35 nodes and 83 edges (Supplementary Figure S6a), with the second most prominent network included 9 nodes and 9 edges (Supplementary Figure S6b). Circular nodes were colored using a continuous mapping color gradient based on Log2FC, ranging from 1.5 to 10 and −1.5 to −10. However, some of the nodes colored purple represent genes with Log2FC > 10 or <−10, respectively.

Further analysis of the upregulated and downregulated DEGs forming functional networks revealed that many of the downregulated genes are involved in promoting cancer cell apoptosis, inhibiting proliferation, and reducing cancer migration (Figure 10b). Downregulation of RPN2 proved to trigger apoptosis and inhibit colorectal cancer migration and invasion [30]. The SSR2 knockdown promoted apoptosis and inhibited the hepatocellular carcinoma HepG2 cells’ migration, invasion, and proliferation in vitro and progression in vivo [31]. The depletion of STT3A significantly reduced the ability of tumor cells to migrate through activation of epithelial-to-mesenchymal transition (EMT) signaling [32]. Dyskerin pseudouridine synthase 1 (DKC1) is highly expressed in multiple types of human cancers, and its depletion arrested the cell cycle and induced senescence, thus inhibiting cancer cells’ migration and proliferation [33]. Downregulation of the heterogeneous nuclear ribonucleoprotein C (HNRNPC) inhibited the invasion and metastasis of hepatocellular carcinoma [34]. STRING developed a confidence score that ranks the associations and interactions from most reliable to least reliable. This ranges between 0 and 1, with scores closer to 1 having a higher estimated likelihood of being true. The functional interactions of the downregulated DEGs in this network (Figure 10b) were obtained with a high confidence > 0.8 cutoff, which greatly increases the likelihood that these DEGs are networking together. Analysis of downregulated DEGs strongly correlated with the most significantly enriched pathways of the GO PEA associated with epithelial tube morphogenesis, Rho protein signal transduction, and regulation of Rho protein signaling (Figure 8a). Altogether, many PsA-induced downregulated genes of interest are interacting with one another to promote apoptosis and reduce tumor cells’ motility.

### 2.19. PsA Effects on the Ubiquitin Proteasome System

The KEGG PEA of the downregulated DEGs showed many enriched pathways involving the 26S proteasome of the UPS. Searching for any of the genes appearing in the functional networks was also implemented in UPS and identified multiple hits. RPN2 proved to dock ubiquitin processing factors and was reported to have multiple interactions with ubiquitin-like domain-containing proteins [35]. The ATP-binding cassette protein A1 (ABCA1) mediated the transfer of phospholipids and circulating cholesterol to apolipoprotein A-1, which then forms high-density lipoprotein (HDL) [36]. ABCA1 is degraded by the proteasome in cholesterol-loaded macrophages through interaction with the COP9 signalosome, a key molecule in controlling protein ubiquitination and deubiquitination [37].

## 3. Discussion

Despite the progress in early PC diagnosis, many diagnosed patients have locally advanced disease and distant metastasis at initial diagnosis. ADT and APIs are initially effective in CSPC suppression, but inevitably, they develop resistance. The mitogenic role of PCSK9 in several malignancies has been recently documented. PsA was validated as a novel orally active dual inhibitor of PCSK9 secretion and PCSK9-LDLR PPI [6,7,8]. PsA has also shown promising in vivo results in nude mouse xenograft models by suppressing BC and mCRPC progression and recurrence in daily oral doses as low as 10 mg/kg [6,7,8]. Initial appraisal of pharmacologically validated hits and leads is a critical endpoint at the early stages of the drug development pipeline. PsA has a definite promising therapeutic potential against mCRPC, coupled with acute single-dose safety results in a murine model, displaying no major organ toxicity at doses up to 500 mg/kg, presenting an initial safety profile for this molecule going forward [9].

Following a 90-day daily oral dosing safety study of up to 80 mg/kg PsA, male and female *Swiss albino* mice fed on HFD showed no significant change in body weight nor abnormalities in their neurological or autonomic responses. Sub-chronic doses of 10 mg/kg, 40 mg/kg, and 80 mg/kg were chosen based on the findings of the Up-and-Down toxicity procedure previously performed by this study team that resulted in an LD_50_ of >550 mg/kg for PsA oral dosing in *Swiss albino* mice [9]. The male 10 and 40 mg/kg dosing groups saw a significant decrease in the heart weights as compared to VC; however, this was not mirrored in the female nor in the male mice 80 mg/kg dosing groups. Knocking out PCSK9 in experimental animals can lead to heart failure with preserved ejection fraction [38]. However, this cardiotoxic effect resulted in an increase in overall heart weight with a significant increase in the diastolic relative wall thickness versus wild-type animal control. Thus, the sex-specific decrease in average heart weight is not cardiotoxicity due to the PCSK9 inhibitory effects of PsA. Chronic drug exposure can potentially lower heart weight through mechanisms like cardiac atrophy, drug-induced oxidative stress, or mitochondrial dysfunction. PsA in vivo testing for effects on oxidative stress and mitochondrial dysfunction might be required to further assess the potential sex-specific long-term cardiac safety.

Elevation of the liver transaminases AST and ALT is a reliable marker of liver injury/necrosis. However, in this study, systemic AST level for both males and females and ALT level for the female mice were significantly lower than those of the VC group. A study screened >12,000 mice and determined the average male AST levels to be ~50 U/L, female AST levels to be ~48 U/L, male ALT levels to be ~26 U/L, and female ALT levels to be ~30 U/L [39]. The slight increase above the baseline levels for AST and ALT in the control mice is likely caused by the HFD use over the study course, inducing a non-alcoholic fatty liver disease (NAFLD), characterized by excessive lipid accumulation in hepatocytes [40]. The most severe form of NAFLD is nonalcoholic steatohepatitis (NASH), which can lead to liver fibrosis/cirrhosis. The increase in liver AST level due to the HFD was more pronounced in the female VC group. Observing the significant decrease in the AST and ALT levels in treatment groups, nearly to the healthy levels, ~48 U/L and ~30 U/L, respectively, demonstrated the PsA potential hepatoprotective effects against the long-term HFD use. The most significant decrease in AST levels was more pronounced in the female treatment groups. PsA possibly protected mouse livers against the buildup of triglycerides by long-term intake of HFD and subsequently reduced the AST and ALT levels back to the baseline levels. While PCSK9 inhibitors are effective for hyperlipidemia, there is inconclusive evidence correlating circulating PCSK9 levels with the development or reversal of NAFLD [41]. The absence of NAFLD features in treated groups clearly indicates the safety and anti-hyperlipidemic uniqueness of PsA.

Elevated levels of BUN or CREAT are common nephrotoxicity indicators. A decrease in BUN levels is usually linked to malnutrition, overhydration, or a low-protein diet. The CREAT levels of both male and female treatment groups showed no significant change over the 90-day dosing regimen (Supplementary Tables S3 and S4). This, paired with the lack of necrosis, inflammation, or other signs of nephrotoxicity in the histopathological examination of the kidney tissue sections, justifies that the decrease in BUN is not due to an overt toxicity on the nephrons (Supplementary Figure S5b).

Hematological parameters remain important markers for the prediction of injury due to xenobiotic exposure in humans and animals. Negative hematological results in animals have a high correlation with similar negative results in humans [42]. The average WBC-H, RBC-H, HGB, HCT, MCHC, and PLT count in PsA-treated mice indicates no signs of infection, inflammation, aplastic or hemolytic anemia, or thrombocytopenia, as the levels were in accordance with literature parameters observed in healthy mice [43]. Only the MCV of the male 40 mg/kg treatment group value of 55.4 ± 4.8 fL was slightly lower, but still well within the reference interval of 57.81 ± 7.79 fL seen in healthy male *Swiss albino* mice [43]. The cause of the missing hematological data for the female 40 mg/kg and 80 mg/kg treatment groups was due to an error in proper blood collection during animal sacrifice, leading to blood samples clotting before analysis. While this lack of data does weaken the safety study, the absence of deleterious effects in the hematological parameters of the male treatment groups up to 80 mg/kg as well as the lack of significant change in any of the hematological parameters between the female control and the 10 mg/kg-treated group suggest the lack of drastic changes in the hematological parameters for the 40 mg/kg and 80 mg/kg female dosing groups. This hypothesis is strengthened by the hematological data collected earlier in the 14-day acute PsA toxicity assessment, which observed healthy levels of WBC-H, RBC-H, HGB, HCT, MCV, MCHC, and PLT count with doses up to 500 mg/kg level [9].

Motivated by the HFD use over the 90-day sub-chronic safety study course, PsA’s ability to reduce plasma cholesterol in treated *Swiss albino* mice was assessed. Daily PsA oral dosing greatly reduced the total plasma cholesterol levels in both female and male treatment groups, relative to the VC. The male mice displayed a greater relative plasma cholesterol reduction response. PsA anti-hypercholesterolemic effect was not only comparable with the FDA-approved marketed PCSK9-LDLR PPI inhibitory humanized mAb evolocumab whose clinical trials reported total cholesterol reduction by −26.5% after 8 dosing weeks, but also was in accordance with a propensity score matching analysis that showed greater relative % reduction for LDL-C in males over females (−54.4% versus −42.7%, *p* < 0.001) [44]. This clearly favors PsA anti-hyperlipidemic validity as an orally active PCSK9-targeting lead and rationalizes its use for the control of PCSK9-sensitive mCRPC.

Development of a sensitive, specific, reproducible, and reliable analytical assay is paramount to assess PKs. An HPLC method was first developed for the detection and separation of PsA from mouse plasma constituents and later optimized for linearity, selectivity, sensitivity, recovery, accuracy, stability, and precision. The developed HPLC analytical method in mouse plasma was in accordance with the guidelines for bioanalytical method validation of the Food and Drug Administration [29]. It is imperative to understand PsA PKs properties before subsequent preclinical testing as a valid anti-PC recurrence lead. Deciding on the IV dosing concentration of 50 mg/kg for the PK study was due to limitations in PsA aqueous solubility and limited maximum IV volume administration in mice. An IP dosing concentration of 125 mg/kg afforded prominent HPLC peaks utilized for the PsA qualitative tissue distribution assessment. Yet, the quantitative distribution of PsA in mouse organs was still unknown. IP dosing allowed higher dosing volumes of PsA without causing harm to the animals. The developed HPLC method was sensitive enough to easily detect much lower PsA concentrations.

Following IV administration, PsA demonstrated rapid distribution and elimination in the mouse body. This was determined using the calculated parameters, including an initial concentration range of 24.6–34.2 µg/mL, an average T_1/2_ of 0.53 h, an average V_d_ of 1.83 L/kg, and an average clearance rate of 2.39 L/h. These parameters fit into a non-compartment model. The notable pharmacodynamics effect of PsA in PC and BC further evidenced its wide distribution into the mouse tissues. The volume of distribution of 1.83 L/kg was significantly higher than the average total body water volume in mice (0.6 L/kg), indicating potentially elevated binding to peripheral tissues [45]. The short half-life of 0.53 h does pose challenges for future druggability and would require frequent doses to be administered. However, knowledge surrounding drug half-life is considered insufficient whenever the tested parent lead has active metabolites that might prolong its activity duration. Although PsA was not detectable in the plasma through the validated HPLC method following oral dosing, it was detectable following IP dosing. This was parallel to notable anticancer pharmacodynamic effects observed in cancer models [6,7,8]. It is thus assumed that PsA is being extensively metabolized by the gut microbiota in the oral route. Future research is needed to uncover PsA active metabolites to further elucidate its pharmaceutical potential.

PsA showed effective mCRPC recurrence suppression in a clinical-mimicking model of nude mice xenografted with the mCRPC CWR-R1ca-Luc exposed to neoadjuvant DTX (3 weeks), ENZ (4 weeks), followed by primary tumor surgical excision. PsA daily oral dosing over 60 days effectively suppressed the mCRPC locoregional and distant recurrences at both 10 mg and 20 mg/kg. Specifically, PsA showed superior bone, lung, liver, and spleen mCRPC distant recurrences suppression versus VC. PsA did not prevent mCRPC brain distant recurrences in both treatment doses versus the VC group, which was in-line with its poor distribution into the brain due to potential failure to cross the BBB.

RNA-sequencing was used to develop a genomic profile for PsA 20 mg/kg treatment effects on the only locoregionally-recurred mCRPC tumor in the aforementioned nude mice model (Table 5). PEA and PPI were subsequently utilized to help identify potential molecular targets, predict disease pathways, and elucidate the underlying therapeutic mechanism of PsA. The KEGG and GO pathway enrichment analysis of the biological processes of the most significantly enriched upregulated and downregulated DEGs displayed strong evidence for neurological disorders, tumor suppression, and anti-migratory effects. Cell junction assembly was identified as the most significantly enriched GO pathway associated with upregulated DEGs (Supplementary Figure S6a). Intercellular adhesion can resist the action of EMT and reduce the invasion potential of cancer cells. Thus, upregulation of genes associated with this pathway following PsA treatment provided strong correlation and mechanistic insights into its PC recurrences suppressive effects observed in previous studies [7,8]. The most significantly enriched GO pathways associated with the downregulated DEGs were epithelial tube morphogenesis, Rho protein signal transduction, and regulation of Rho protein signal transduction (Figure 8a). During EMT, epithelial cells are converted into more active and invasive mesenchymal cells and lose their characteristics that promote differentiation [46]. This includes cell-cell adhesion, which correlates with the most significantly enriched GO pathway associated with the upregulated genes. The Rho GTPases facilitate a wide range of malignant cellular processes, including cell migration, survival, and proliferation [47]. Downregulation of Rho GTPases provides further mechanistic justification for the PsA anti-PC effects.

KEGG PEA of the downregulated genes resulted in eight enriched canonical pathways associated with 26S proteasome-mediated protein degradation. The 26S proteasome is widely known to be the major degradative machinery of the UPS, which is the cell’s foremost protein degradation system [13,14,15,16,17]. The UPS is responsible for the bulk of protein degradation at the endoplasmic reticulum (ER).

A substantial number of cell, animal, and human studies have correlated the disruption of the UPS as a primary or secondary source in the pathogenesis of numerous neurodegenerative conditions [13,14,15,16,17]. The PEA also mapped multiple downregulated genes to the assembly and trafficking of telomerase. Downregulated telomerase can cause telomere attrition, which may be linked to the development of neurodegenerative diseases [13,14,15,16,17]. However, data analyses of PsA acute and chronic safety studies did not show any prominent neurodegenerative markers in the brain histopathology, such as Lewy bodies. There were also no changes in behavioral, autonomic, or neurological responses in either study that would hint at neurotoxicity. PsA failed to cross the BBB and was not detectable in the brain, though it was detected in four other organs following a single 125 mg/kg IP dosing (Figure 6). These observations provided compelling evidence for PsA’s lack of potential neurotoxicity. Since the RNA-Seq data were acquired on recurrent mCRPC tumors and not normal nervous or neuronal tissues, the anticancer effects associated with the downregulation of UPS-related genes hold more potential than the neurotoxic effects. Multiple UPS inhibitors have gained FDA approval for the control of multiple myeloma and non-solid malignancies.

Analyses of functional networks from the PPIs of upregulated and downregulated DEGs was in line with the results from the PEA and exhibited gene/protein networks with researched associations to potential neurological disorders and anticancer effects. Analysis of downregulated DEGs resulted in networks and pathways that showed direct and indirect associations with the UPS. Cancer cells are more dependent on the upregulated UPS than normal cells to degrade the tumor suppressor proteins and prevent apoptosis [48]. Downregulation of UPS can diminish cancer cells’ ability to respond to stress by inhibiting ubiquitination at sites related to oncogenic pathways [49]. Downregulation of the UPS by PsA treatments cannot reduce efficiency in degrading damaged and misfolded proteins in the brain, nor can it raise DEGs neurodegenerative markers, since it will not cross BBB. Furthermore, the UPS was proven to be implemented in the progression of the CSPC-to-CRPC phenotypes with predominance in the mCRPC [21,24]. The UPS and specifically the 26S proteasome complex is an emerging molecular target for cancer therapy. This study shows that PsA might uniquely inhibit the UPS in recurrent mCRPC, in addition to targeting the PCSK9-LDLR axis. Dual targeting of the PCSK9-LDLR axis and UPS by PsA is a novel mCRPC-controlling strategy.

This is the first study to report the sub-chronic safety, PKs, biodistribution, and transcriptomics of PsA using murine models. This initial preclinical assessment data highlights the PsA potential as a novel mCRPC recurrence suppressor lead.

## 4. Materials and Methods

### 4.1. Experimental Animals

Twenty male athymic nude mice (Strain Foxn^nu^-Foxn^1+^), 7–8 weeks old, were used for the therapeutic recurrence model (Protocol # 21DEC-KES-01). Sixteen male and fourteen female *Swiss albino* mice, 19–20 weeks old, were used for PKs and distribution studies (Protocol # 21DEC-KES-02). Twenty male and twenty female *Swiss albino* mice, 7–8 weeks old, were used for a chronic toxicity study (Protocol # 21DEC-KES-03). Mice were purchased from Envigo (Indianapolis, IN, USA). The animals were given a 2-week acclimatization period and maintained under clean room conditions with a relative humidity of 55–65%, a temperature of 22 ± 2 °C, a 12:12 h light/dark cycle, Alpha-Dri bedding, and free access to drinking water and pelleted rodent chow. Animals were housed in group cages (male, *n* = 5; and female, *n* = 5) and kept in the same environmental conditions. All animal experiments were approved by the Institutional Animal Care and Use Committee (IACUC), University of Louisiana at Monroe, and were conducted in strict accordance with good animal practice as defined by the National Institutes of Health (NIH) guidelines.

### 4.2. Systemic Cholesterol Monitoring Assay

Plasmatic cholesterol concentrations in mice were determined using a total cholesterol kit (EnzyChrom^TM^ Cholesterol Assay Kit ECCH-100 BioAssay Systems, Hayward, CA, USA), adhering to the manufacturer’s instructions. Colorimetric quantitation was measured at 340 nm using a Synergy 2 microplate reader (BioTek Instruments Inc., Winooski, VT, USA). Blood was collected through tail vein extraction on the day before treatment, day 35, and day 90. Blood was quickly collected into green-topped Lithium Heparin 400 µL blood collection tubes (BD Microtainer REF# 365965 Becton, Dickinson and Company, Franklin Lakes, NJ, USA), centrifuged at 13,000× *g* for 10 min, and the upper layer of plasma was transferred to 1.5 mL Eppendorf tubes (Thermo Fisher Scientific, Waltham, MA, USA) and stored at −20 °C until analysis.

### 4.3. Chemicals and Reagents

HPLC grade CH_3_CN (Millipore–Sigma, Darmstadt, Germany) and 18 Ω water (PHARMCO-AAPER, Brookfield, CT, USA) containing 0.1% formic acid (Millipore–Sigma, Darmstadt, Germany) were used for the mobile phase. 2-Benzofuran carboxylic acid purchased from Aldrich Chemical Company Inc. (Milwaukee, WI, USA) was used as IS. PsA was extracted from a 14-day *Aspergillus fumigatus* fermentation broth [6,7,8]. It was then further isolated on Sephadex LH20 using CH_2_Cl_2_-EtOAc (VWR Chemicals BDH, Radnor, PA, USA) gradient elution and finally purified on LiChroprep RP-18, 25–40 µm, C-18 RP (Millipore–Sigma, Darmstadt, Germany) gravity column using isocratic 30% CH_3_CN in H_2_O (PHARMCO-AAPER, Brookfield, CT, USA) to a purity of >99% guided by HPLC and q^1^H NMR. EtOAc and isopropanol purchased from Avantor Science Central (Allentown, PA, USA) were used in the extraction of PsA from mouse plasma. Dried remains were reconstituted in HPLC-grade methanol (PHARMCO-AAPER, Brookfield, CT, USA).

### 4.4. The 90-Day Sub-Chronic Toxicity Study Design

For the 90-day daily oral dosing sub-chronic toxicity study, twenty male and twenty female healthy 8-week-old *Swiss albino* mice were randomly selected and randomized to 8 groups (*n* = 5/sex/group). Mice received free access to purified drinking water and high-fat diet (HFD) pelleted rodent chow (11.4% total fat, no. 7004, Envigo/Teklad, Madison, WI, USA) until the day before sacrifice, at which time they were fasted for 8 h and allowed only free access to drinking water. Daily doses of PsA (10, 40, 80 mg/kg body weight) were administered to the mice formulated in <5% DMSO (Mediatech Inc., Manassas, VA, USA) and <0.2% Tween 80 (Croda Inc., Princeton, NJ, USA) at a volume not exceeding 1% of body weight by metal oral gavage (2 mm diameter with stainless steel bite protector, 18-gauge, 3.81 cm long). The mice were observed for mortality, morbidity, and behavioral signs of pain/toxicity at 1, 2, 4, 6, 12, and 24 h and then once every subsequent day for a total of 14 days. The mice were observed weekly until the completion of the study. The body weights of the mice were measured in grams using a Scout^TM^ Pro (Ohaus Corp., Pine Brook, NJ, USA) on day 0, 4, 8, 11, 14, 21, 28, 35, 42, 49, 56, 63, 70, 77, 83, and 90 after initial treatment. On day 90, all mice were anesthetized using isoflurane (USP-vaporizer method, Matrix VIP-3000, Covetrus, Dublin, OH, USA, exposure of animals in an anesthesia chamber at 3% isoflurane vaporization rate. Exposure time 2 min). Mice were then euthanized by cervical dislocation according to the 2020 AVMA Guidelines on Euthanasia and dissected [50]. Mice organs (brain, liver, kidney, heart, lungs, spleen) were excised and weighed for histopathological examination. The organs were stored in 10% neutral buffered formalin (Avantor Science Central, Allentown, PA, USA) for 24 h and then transferred to 70% ethanol (AAPER ALCOHOL, Shelbyville, KY, USA). This study was performed following the OECD guideline 408 for the testing of chemicals [51].

#### 4.4.1. Hematological and Biochemical Evaluation

On the day of sacrifice, mice were decapitated, and the blood was quickly collected into a green-topped Lithium Heparin 400 µL blood collection tube (BD Microtainer REF# 365965) as well as a purple-topped K2 EDTA (K2E) 500 µL blood collection tube (BD Microtainer, Cat# 365974) to ensure all blood samples were collected free of clots. The Li Heparin samples were immediately centrifuged at 13,000× *g* for 10 min, and the plasma was transferred to 1.5 mL Eppendorf tubes free of Li heparin for biochemical analysis. Plasma samples were analyzed for AST, ALT, ALP, glucose, BUN, and creatinine levels using the Beckman AU680 clinical chemistry analyzer system (Beckman Coulter, Atlanta, GA, USA). The ETDA samples were analyzed for hematological parameters, and WBC, RBC, Hgb, Hct, MCV, MCHC, and PLT were determined using the Siemens Advia 120 hematology analyzer (Siemens Healthcare Diagnostics Inc., Tarrytown, NY, USA). All blood samples were analyzed at the LSU School of Veterinary Medicine Clinical Pathology Laboratory at Baton Rouge, Louisiana.

#### 4.4.2. Paraffin Embedding and Staining

The experimental animals’ organs were carefully excised and quickly fixed in 10% neutral buffered formalin for 24 h before being transferred to 70% ethanol. The organs were embedded in paraffin before sectioning and staining with hematoxylin and eosin (H&E). All sectioning and staining were conducted at the AML Laboratories (Jacksonville, FL, USA). The paraffin-embedded tissues were sliced into 5 µm-thick sections before being mounted on a positively charged slide. The 5 µm-thick sections were then dewaxed using xylene, rinsed using 80–95% ethanol, and rehydrated using H_2_O. They were subsequently stained with H&E before being dehydrated again using 80–95% ethanol to xylene.

#### 4.4.3. Statistical Analysis

Results for the hematological and biochemical parameters were analyzed separately by One-way Analysis of Variance (ANOVA), followed by multiple comparisons with Dunnett’s test. All statistical analyses were performed using GraphPad Prism version 8 software (San Diego, CA, USA). Data in this study were expressed as a mean ± SD (standard deviation) and mean ± SEM (standard error of mean). A probability value of <0.05 was considered statistically significant (* *p* < 0.05; ** *p* < 0.01; and *** *p* < 0.001).

### 4.5. HPLC and Chromatographic Conditions

Chromatographic analysis was conducted on a Shimadzu HPLC system (Shimadzu USA Manufacturing Inc., Canby, OR, USA) equipped with an SPD-20A UV detector, 20 µL manual sampler, LC-20AD pump, a degasser, and an automated temperature-controlled column compartment. The UV detector, column oven, and LC-20AD pump were coupled to the Shimadzu system and controlled through Lab Solutions software, v5.111. Stationary phase separation was carried out using a Shimadzu Shim-Pack GWS C18 column (4.6 × 250 mm, 5 µm) equipped with a Zorbax ODS guard column (4.6 × 12.5 mm, 5 µm) by gradient elution with the column temperature maintained at 35 °C. The UV detector wavelength was set and measured at 254 nm. A constant flow rate of 1.0 mL/min and manual injection of 20 µL were employed throughout the HPLC analysis. A mobile phase consisting of aqueous HCOOH (0.1%, *v*/*v*, mobile system A) and HPLC grade CH_3_CN (mobile system B) utilized with a gradient elution of 30–60% B at 0–5 min, 60–85% B at 5–8 min, 85–95% B at 8–11.5 min, and 95% B at 11.5–18 min. The post-run re-equilibration time was 5 min.

#### 4.5.1. Calibration Standard and Sample Preparation

For preparation of the stock solution, 1.7 mg of >99% pure PsA was weighed and dissolved in 1 mL HPLC-grade methanol. Subsequent dilutions were carried out using 18 Ω water to reach standard solutions of 50, 40, 30, 20, 10, 8, and 4 µg/mL PsA. They were stored at −20 °C and used within 4 h for analysis. 2.0 mg of 2-benzofuran carboxylic acid internal standard (IS) was weighed and dissolved in 1 mL HPLC-grade methanol before being diluted using 18 Ω water to a working concentration of 50 µg/mL. It was stored at −20 °C until use throughout HPLC analyses. For the intravenous (IV) and intraperitoneal (IP) injections, ~5 mg, respectively, of >99% pure PsA was dissolved in 15 µL DMSO before being combined with 230 µL sterilized phosphate buffer saline (PBS) and 5 µL Tween 80 to make a stock volume of 250 µL. The PsA concentration injected was 50 mg/kg for IV and 125 mg/kg for IP dosing based on each mouse’s individual weight.

#### 4.5.2. PsA Extraction Method

Five µL of the IS (50 µL/mL) and 20 µL of 50, 40, 30, 20, 10, 8, and 4 µg/mL PsA standard solutions were combined with 15 µL freshly extracted blank mouse plasma. 300 µL of a 70% EtOAc/30% isopropanol mixture was added and vortexed for 30 s. Following centrifugation at 15,000× *g* for 10 min, the supernatant was transferred to a 2 mL Eppendorf tube for nitrogen evaporation. The residues were reconstituted in 25 µL HPLC-grade MeOH before being subjected to 30 s sonication followed by centrifugation at 15,000× *g* for 10 min. Afterwards, the entire sample was immediately injected for RP-HPLC analysis. The mouse plasma samples used in the PK study were subjected to the same extraction method, minus the addition of the PsA standard solutions. After weighing, each mouse organ was reconstituted in 500 µL PBS, then homogenized using the MISONIX Sonicator (Division of QSonica LLC, Newtown, CT, USA). Samples were centrifuged at 15,000× *g* for 10 min, and the supernatant was collected and transferred to a 2 mL Eppendorf. About 5 µL of IS (25 µL/mL) and 300 µL of a 70% EtOAc/30% isopropanol mixture were added to 100 µL of supernatant and vortexed for 30 s. Following centrifugation at 15,000× *g* for 10 min, the supernatant was transferred to a 2 mL Eppendorf tube and evaporated under N_2_. The remains were reconstituted in 40 µL HPLC-grade methanol before being subjected to 30 s sonication followed by centrifugation at 15,000× *g* for 10 min.

#### 4.5.3. Method Validation

The HPLC method validation was conducted for linearity, selectivity, sensitivity, recovery, accuracy, stability, and precision of PsA in mouse plasma in accordance with the guidelines for Bioanalytical Method Validation of the Food and Drug Administration [29]. Calibration curves were constructed using the peak-area ratios of the PsA analyte to the internal standard vs. plasma concentrations. Quality control (QC) samples at four concentrations (4, 32, 50, and 250 µg/mL) were used to assess method validation.

#### 4.5.4. Linearity

A calibration curve prepared from seven calibration points by linear regression with a weighting factor of 1/x was used to quantify the concentration of PsA in mouse plasma samples. A ratio for analyte concentration was determined by plotting analyte/IS peak area under the curve, and the linear calibration equation with its correlation coefficient (r) was determined.

#### 4.5.5. Accuracy and Precision

The intra-day precision and accuracy were evaluated by analyzing fresh mouse plasma spiked with respective concentrations at three replicates within the same day. Inter-day precision and accuracy were evaluated in the same way for four consecutive days (*n* = 3). The precision was calculated using the coefficient of variation (CV) for the analysis of QC samples. The CV for each QC did not deviate by more than ±15%, in accordance with the guidelines for Bioanalytical Method Validation of the FDA [29].

#### 4.5.6. Stability

The stability of PsA at room temperature was determined by preparing six separate mouse plasma samples for each concentration. Three samples for each concentration were immediately extracted and injected into the HPLC. The three remaining samples were left on a lab bench protected from light for 24 h before being extracted and injected into the HPLC. The concentrations measured at the 0 and 24 h time points were compared. The stability of PsA after three freeze-thaw cycles was assessed by preparing six separate mouse plasma samples for each concentration. Three samples for each concentration were immediately extracted and injected into the HPLC. The three remaining samples for each concentration were frozen and thawed in a −80 °C freezer three separate times before being extracted and injected into the HPLC. The stability of PsA at −20 °C for 2 weeks was determined by preparing six separate mouse plasma samples for each concentration. Three samples for each concentration were immediately extracted and injected into the HPLC. The three remaining samples were left in a −20 °C freezer for two weeks before being thawed, extracted, and analyzed on the HPLC.

#### 4.5.7. Recovery

Recovery of PsA was determined by comparing the mean peak area under the curve (AUC) obtained from the extracted mouse plasma with the AUC obtained by the direct injection of the corresponding spiked standard solutions.

#### 4.5.8. Pharmacokinetics Analysis

The mean plasma concentration-time curve was plotted using GraphPad Prism software version 8.0.2 (La Jolla, CA, USA). PK parameters were obtained through non-compartmental analysis following intravenous bolus input using PK Solver 2.0 [52].

### 4.6. PsA PKs Study in Swiss Albino Mice

For the PKs study, twelve male and twelve female healthy *Swiss albino* mice were randomly selected and placed in 3 groups (*n* = 4/sex/group). The mice received free access to drinking water and pelleted regular rodent chow (5% fat content, Cat# 7012, Envigo/Teklad, Madison, WI, USA) until the day before experimentation, after which they were fasted for 8 h and allowed only free access to drinking water. Mice were administered PsA formulated in sterile PBS-DMSO (925:75) + 0.2% Tween 80 at a volume not exceeding 100 µL intravenously through recto-orbital injection. Each animal was anaesthetized using Isoflurane (Piramal Critical Care Inc., Bethlehem, PA, USA) for 1–2 min before injection. Disposable sterilized syringes (Becton, Dickinson and Company, Franklin Lakes, NJ, USA) were used for blood collection as well as administration of PsA at a dose of 50 mg/kg and volume, adjusted to the mouse body weight, no greater than 100 µL for animal safety considerations. Blood samples of around 50–70 µL were collected through the tail vein in 600 µL Eppendorf tubes at time points 0.25, 0.75, and 1.5 h for groups 1 and 4, 0.083, 0.5, and 1 h for groups 2 and 5, in addition to 2 and 4 h for groups 3 and 6 following intravenous injection and subjected to immediate centrifugation. From the plasma, 15 µL was transferred into clean 600 µL Eppendorf tubes and immediately subjected to the extraction method listed above and analyzed using HPLC.

### 4.7. PsA Biodistribution Study in Swiss Albino Mice

For the biodistribution study, four healthy male and two healthy female *Swiss albino* mice were randomly selected and placed in 2 groups (*n* = 3/group). The mice received free access to drinking water and pelleted regular rodent chow (Cat# 7012, Envigo/Teklad, Madison, WI, USA) until the day before experimentation, after which they were fasted for 8 h and allowed only free access to drinking water. One group was IP administered 125 mg/kg PsA formulated in PBS-DMSO (925:75) + 0.2% Tween 80 at a body weight-adjusted volume not exceeding 300 µL. Mice were then sacrificed through cervical dislocation 7 min after injection. Disposable sterilized syringes (Becton, Dickinson and Company, Franklin Lakes, NJ, USA) were used for the injection. The blood was collected in 600 µL Eppendorf tubes. The brain, liver, kidney, spleen, and heart were excised, weighed, and homogenized using the MISONIX Sonicator (Division of QSonica LLC, Newtown, CT, USA). Collected samples were immediately extracted using the described method above and analyzed using HPLC.

### 4.8. The mCRPC Recurrence Prevention Study in Athymic Nude Mice

For the recurrence prevention study, nineteen 7–8-week-old athymic male nude mice (Strain Foxn^nu^-Foxn^1+^) were xenografted with 2 × 10^6^ the mCRPC CWR-R1ca-Luc cells at the suprascapular region in 100 µL 1:1 serum-free Dulbecco’s Modified Eagle Medium (DMEM) medium-Matrigel. Mice were fed on an HFD (11.4% total fat, Cat# 7004, Envigo/Teklad, Madison, WI, USA) for 7 days before the tumor cells xenografting. When the tumors reached 100 mm^3^, mice were subjected to weekly IV injections of 10 mg/kg docetaxel (DTX) (Combi-Blocks, San Diego, CA, USA) dissolved in Tween 80 (20 mg/mL) for four consecutive weeks, followed by daily IP injections of 5 mg/kg ENZ (Combi-Blocks, San Diego, CA, USA) in sterile PBS for three weeks. Following these neoadjuvant regimens, mice were randomly selected and placed into three groups. Groups 1 (*n* = 7) received daily oral doses of 20 mg/kg PsA (prepared in sterile PBS-DMSO 925:75). Group 2 (*n* = 5) received daily oral PsA 10 mg/kg. Group 3 *(n* = 7) received daily oral doses of PsA-free vehicle control. Treatments continued for 60 days. Isoflurane-anesthetized mice were subjected to weekly tumor monitoring using PerkinElmer IVIS Lumina Series III (Waltham, MA, USA) 30 min after an IP injection of (+)-luciferin (75 mg/kg, IP, PerkinElmer, Waltham, MA, USA) for efficiency assessment of excision surgery, tumor progression, and tumor metastasis. Following treatment regimen completion, mice were anesthetized using isoflurane (USP-vaporizer method, Matrix VIP-3000), exposure of animals in an anesthesia chamber at 3% isoflurane vaporization rate. Exposure time 2 min. Mice were then euthanized by cervical dislocation according to the American Veterinary Medical Association (AVMA) Guidelines for the Euthanasia of Animals: 2020 Edition, and tumors were collected [50]. Tumors were snap frozen using Isopentane (J.T. Baker, Radnor, PA, USA) in liquid nitrogen before storage at −80 °C.

### 4.9. Tumor RNA Extraction

Following spraying hands with RNaseZap^TM^ (Invitrogen Thermo Fisher Scientific, Waltham, MA, USA), excised tumor cells from the mCRPC recurrence prevention study were defrosted from −80 °C and 85–95 mg were weighed and transferred to a 1.7 mL RNase/DNase-free Eppendorf (Genesee Scientific, Morrisville, NC, USA). About 1 mL of TRizol (Invitrogen-Thermo Scientific, Waltham, MA, USA) was added to each Eppendorf before being homogenized using a MISONIX Sonicator (Division of QSonica LLC, Newtown, CT, USA). Afterwards, samples were stored on ice for 2 h. About 200 µL of molecular-grade CHCl_3_ (ThermoFisher Scientific, Waltham, MA, USA) was added to each Eppendorf and incubated for 3 min at room temperature. Samples were vortexed for 30 s, then put on ice for 15 min to allow for phase separation. The upper layer was then collected and centrifuged at 4 °C and 12,000× *g* for 15 min. The supernatant was collected, and 500 µL of molecular-grade isopropyl alcohol (ThermoFisher Scientific, Waltham, MA, USA) was added before incubation on ice for 30 min. Samples were centrifuged at 4 °C and 12,000× *g* for 10 min, isopropyl alcohol was decanted, and the RNA pellet was reconstituted with 1 mL 70% molecular grade ethanol (Decon Labs, King of Prussia, PA, USA). Samples were then centrifuged at 4 °C and 12,000× *g* for 10 min, the ethanol supernatant was removed, and an ethanol wash was repeated twice more. RNA pellet was allowed to air dry for 5 min to remove excess traces of ethanol before being suspended in 20–50 µL RNase-free water (VWR International, LLC, Radnor, PA, USA). Samples were stored in −80 °C freezer until the next day, when they were measured using a Reichert (Reichert Technologies, Depew, NY, USA) nanodrop. Nearly 1000 ng were prepared and submitted for sequencing analysis.

#### 4.9.1. RNA-Seq Data Collection and DEG Identification

RNA-Sequencing was performed at a strand-specific 100-cycle paired-end resolution, in an Illumina NovaSeq 6000 sequencing machine (Illumina, San Diego, CA, USA). The samples were multiplexed in two lanes of a flow-cell, resulting in between 48.71 and 40.04 million reads per sample. The read quality was assessed using the FastQC software, v0.12.0 [53]. On average, the per-sequence quality score measured in the Phred quality scale was above 30 for all the samples. As expected for RNA-sequencing data, QC did not reveal the presence of adapters that required trimming in the sequenced reads. The reads were mapped to the joint human (GRCh38) and mouse (GRCm39) genomes using the STAR software, version 2.7.11b [54]. On average, 91% of the sequenced reads mapped to the combined genome, resulting in between 42.9 and 36.0 million mapped reads per sample, of which, on average, 80.4% were uniquely mapped reads. Transcript abundance estimates were calculated using the FeatureCounts software, v2.07 [55]. Expression normalization and differential gene expression calculations were performed using the DESeq2 software, v1.48.2, to identify statistically significant differentially expressed genes [56]. The significance *p*-values were adjusted for multiple hypothesis testing by the Benjamini and Hochberg method, establishing a false discovery rate (FDR) for each gene [57]. The cut-off criteria for assessing the DEGs were log2FC ≥ 1.5 or ≤−1.5, adjusted *p*-value ≤ 0.05, and an FDR ≤ 0.1.

#### 4.9.2. RNA-Seq Data Analysis

Statistical software R (version 4.2.3, https://www.r-project.org) was implemented in the statistical calculation and interpretation of the DEGs. Pathway enrichment analysis (PEA) was performed using the gene set enrichment analysis (GSEA) database [58]. The Gene Ontology (GO) and Kyoto Encyclopedia of Genes and Genomes (KEGG) Medicus gene sets were downloaded from the GSEA database and utilized for the PEA [59,60,61]. The Bioconductor packages, v3.21, clusterProfiler, v4.12.6, enrichR, v1.24.4 and DOSE were used to perform the hierarchical clustering and enrichment analysis of the DEGs [62,63,64,65,66,67]. Biological processes, cellular components, and molecular functions of the DEGs were explored using GO term enrichment analysis. Pathway enrichment analysis of the DEGs was performed using KEGG Medicus. Data visualizations were created using packages in tidyverse [68].

#### 4.9.3. Protein-Protein Interaction and Gene Regulatory Network Analysis

Both lists of upregulated and downregulated DEGs were separately uploaded and analyzed using the STRINGapp in Cytoscape Software, v3.10.4 [69,70]. A confidence cutoff of >0.8 was implemented. This confidence indicator ranges from 0 to 1, with 1 being the highest, and indicates how likely STRING judges an interaction to be true, based on evidence from genomic context, gene co-expression, interaction experiments, molecular pathways, automatic text mining, and curated knowledge.

## Figures and Tables

**Table 1:** Recovery of PsA from mice plasma (*n* = 3).

Concentration (µg/mL)	Recovery	
	Accuracy (%)	CV (%)
4	90.6	18.65
50	116.3	12.61
250	102.0	2.30

**Table 2:** Chemical stability of PsA in mice plasma (*n* = 3).

Concentration (µg/mL)	24 h Room Temperature		Freeze-Thaw Cycles		−20 °C for 2 Weeks	
	Accuracy (%)	CV (%)	Accuracy (%)	CV (%)	Accuracy (%)	CV (%)
4	35.2	8.86	94.0	13.44	83.6	25.65
50	35.5	9.13	108.1	9.92	93.5	29.18
250	48.8	16.34	100.6	3.39	90.6	26.42

**Table 3:** Intra-day and inter-day accuracy and precision of PsA (*n* = 3).

Concentration (µg/mL)	Intra-Day		Inter-Day	
	Accuracy (%)	Precision (%)	Accuracy (%)	Precision (%)
4	85.2	3.36	99.7	8.19
32	96.4	3.01	91.2	4.8
50	94.5	3.2	86.4	3.54
250	85.3	1.98	82.7	5.14

**Table 4:** PK parameters of PsA after a single IV 50 mg/kg dose administered to 24 healthy *Swiss albino* mice following 8 h fasting.

Parameters	Mean (50 mg/kg)
C_0_ (µg/mL)	37.32
T_1/2_ (h)	0.53
AUC_0–t_ (µg h/mL)	19.06
AUC_t–∞_ (µg h/mL)	20.90
AUMC (µg h/mL)	15.41
MRT (h)	0.74
Cl (L/h)	2.39
V_d_ (L)	1.83

C_0_, initial plasma concentration; T_1/2_, elimination half-life; AUC_0–t_, area under the concentration—time curve from zero to the last measurable plasma concentration; AUC_t–∞_, area under the concentration—time cure from zero to infinity; AUMC, area under the moment curve; MRT, mean residence time; Cl, clearance; V_d_, volume of distribution. Pharmacokinetic parameters were determined through non-compartmental analysis of mean plasma PsA concentration at time points 0.083, 0.25, 0.5, 0.75, 1, 1.5, and 2 h utilizing PK Solver 2.0 (*n* = 7).

**Table 5:** The mCRPC recurrence-suppressive activity of PsA in a clinical-mimicking nude mouse xenograft model.

Organ/Tumor	PsA 10 mg/kg ^a^	PsA 20 mg/kg ^a^	VC ^a^
Locoregional recurrence	1/5	1/7	6/7
Femur	1/5	1/7	6/7
Backbone	1/5	0/7	6/7
Lung	1/5	0/7	4/7
Spleen	1/5	0/7	2/7
Heart	1/5	0/7	2/7
Brain	2/5	2/7	2/7
Liver	1/5	1/7	6/7
Kidney	0/5	0/7	2/7

^a^ Male nude mice xenografted with CWR-R1ca mCRPC cells, once the tumor became palpable, animals were subjected to neoadjuvant docetaxel (DTX) followed by enzalutamide (ENZ) treatment regimens as detailed in Materials and Methods. Mice subjected to primary tumors surgical excision, and then divided into three groups treated as follows: PsA 10 mg/kg (*n* = 5), PsA 20 mg/kg (*n* = 7), and vehicle control (VC, *n* = 7). At the study conclusion, animals and their collected organs were subjected to bioluminescence imaging to detect tumor locoregional and distant recurrences, which translated to number values of affected mice per treatment group.

## Data Availability

The RNA-Seq data used to support the findings of this study can be made available by the corresponding author upon request.

## Supplementary Materials

The following supporting information can be downloaded at https://www.mdpi.com/article/10.3390/molecules30193963/s1, Supplementary Figure S1: The 90-day daily oral PsA treatment effects on body weight of male *Swiss albino* mice. Supplementary Figure S2: The 90-day daily oral PsA treatment effects on body weight of female *Swiss albino* mice. Supplementary Figure S3: Effects of PsA treatments on plasma ALT levels in male *Swiss albino* mice. Supplementary Figure S4. Effects of PsA treatments on mean corpuscular volume in male *Swiss albino* mice. Supplementary Figure S5. Histopathological examination of *Swiss albino* female mice treated with differing concentrations of PsA in a 90-day oral dose chronic toxicity study. Supplementary Figure S6. STRING functional network analysis of upregulated DEGs comparing the PsA versus VC-treated CWR-R1ca tumor cells. Supplementary Table S1. Relative organ weight of male *Swiss albino* mice in PsA chronic toxicity study. Supplementary Table S2. Relative organ weight of female *Swiss albino* mice in PsA chronic toxicity study. Supplementary Table S3. Biochemical parameters glucose (GLU), aspartate aminotransferase (AST), alanine aminotransferase (ALT), alkaline phosphatase (ALP), blood urea nitrogen (BUN), and creatinine (CREAT) (mean + SEM) of male *Swiss albino* mice in a chronic toxicity study of PsA. Supplementary Table S4. Biochemical parameters GLU, AST, ALT, ALP, BUN, and CREAT of female *Swiss albino* mice in a chronic toxicity study of PsA. Supplementary Table S5. Hematological parameters of male *Swiss albino* mice in a chronic toxicity study of PsA. Supplementary Table S6. Hematological parameters of female *Swiss albino* mice in a chronic toxicity study of PsA.

## References

[1] Siegel R.L., Kratzer T.B., Giaquinto A.N., Sung H., Jemal A. (2025). Cancer Statistics, 2025. CA Cancer J. Clin..

[2] Škara L., Huđek Turković A., Pezelj I., Vrtarić A., Sinčić N., Krušlin B., Ulamec M. (2021). Prostate Cancer-Focus on Cholesterol. Cancers.

[3] Stopsack K.H., Gerke T.A., Andrén O., Andersson S.O., Giovannucci E.L., Mucci L.A., Rider J.R. (2017). Cholesterol Uptake and Regulation in High-Grade and Lethal Prostate Cancers. Carcinogenesis.

[4] Ajoolabady A., Pratico D., Mazidi M., Davies I.G., Lip G.Y.H., Seidah N., Libby P., Kroemer G., Ren J. (2025). PCSK9 in Metabolism and Diseases. Metabolism.

[5] Vašíček O., Fedr R., Skoroplyas S., Chalupa D., Sklenář M., Tharra P.R., Švenda J., Kubala L. (2020). Natural Pseurotins and Analogs Thereof Inhibit Activation of B-cells and Differentiation into The Plasma Cells. Phytomedicine.

[6] Abdelwahed K.S., Siddique A.B., Mohyeldin M.M., Qusa M.H., Goda A.A., Singh S.S., Ayoub N.M., King J.A., Jois S.D., El Sayed K.A. (2020). Pseurotin A as A Novel Suppressor of Hormone Dependent Breast Cancer Progression and Recurrence by Inhibiting PCSK9 Secretion and Interaction with LDL Receptor. Pharmacol. Res..

[7] Abdelwahed K.S., Siddique A.B., Qusa M.H., King J.A., Souid S., Abd Elmageed Z.Y., El Sayed K.A. (2021). PCSK9 Axis-Targeting Pseurotin A as a Novel Prostate Cancer Recurrence Suppressor Lead. ACS Pharmacol. Transl. Sci..

[8] Abdelwahed K.S., Siddique A.B., Ebrahim H.Y., Qusa M.H., Mudhish E.A., Rad A.H., Zerfaoui M., Abd Elmageed Z.Y., El Sayed K.A. (2023). Pseurotin A Validation as A Metastatic Castration-Resistant Prostate Cancer Recurrence-Suppressing Lead via PCSK9-LDLR Axis Modulation. Mar. Drugs.

[9] McGehee O.C., Ebrahim H.Y., Rad A.H., Abdelwahed K.S., Mudhish E.A., King J.A., Helal I.E., Meyer S.A., El Sayed K.A. (2023). Towards Developing Novel Prostate Cancer Recurrence Suppressors: Acute Toxicity of Pseurotin A, an Orally Active PCSK9 Axis-Targeting Small-Molecule in *Swiss albino* Mice. Molecules.

[10] Jaafar A.K., Techer R., Chemello K., Lambert G., Bourane S. (2023). PCSK9 and the Nervous System: A No-Brainer?. J. Lipid Res..

[11] Rosoff D.B., Bell A.S., Jung J., Wagner J., Mavromatis L.A., Lohoff F.W. (2022). Mendelian Randomization Study of PCSK9 and HMG-CoA Reductase Inhibition and Cognitive Function. J. Am. Coll. Cardiol..

[12] Tan P., Wei S., Yang L., Tang Z., Cao D., Liu L., Lei J., Fan Y., Gao L., Wei Q. (2016). The Effect of Statins on Prostate Cancer Recurrence and Mortality after Definitive Therapy: A Systematic Review and Meta-Analysis. Sci. Rep..

[13] Zelcer N., Hong C., Boyadjian R., Tontonoz P. (2009). LXR Regulates Cholesterol Uptake through Idol-Dependent Ubiquitination of the LDL Receptor. Science.

[14] Chen Y., Wang H., Yu L., Yu X., Qian Y.W., Cao G., Wang J. (2011). Role of Ubiquitination in PCSK9-Mediated Low-Density Lipoprotein Receptor Degradation. Biochem. Biophys. Res. Commun..

[15] Wang Y., Huang Y., Hobbs H.H., Cohen J.C. (2012). Molecular Characterization of Proprotein Convertase Subtilisin/Kexin type 9-Mediated Degradation of the LDLR. J. Lipid Res..

[16] Li Y., Li S., Wu H. (2022). Ubiquitination-Proteasome System (UPS) and Autophagy Two Main Protein Degradation Machineries in Response to Cell Stress. Cells.

[17] Popovic D., Vucic D., Dikic I. (2014). Ubiquitination in Disease pathogenesis and Treatment. Nat. Med..

[18] Scalzulli E., Grammatico S., Vozella F., Petrucci M.T. (2018). Proteasome Inhibitors for The Treatment of Multiple Myeloma. Expert Opin. Pharmacother..

[19] Tang Y., Geng Y., Luo J., Shen W., Zhu W., Meng C., Li M., Zhou X., Zhang S., Cao J. (2015). Downregulation of Ubiquitin Inhibits the Proliferation and Radioresistance of Non-Small Cell Lung Cancer Cells In Vitro and In Vivo. Sci. Rep..

[20] Soave C.L., Guerin T., Liu J., Dou Q.P. (2017). Targeting the Ubiquitin-Proteasome System for Cancer Treatment: Discovering Novel Inhibitors from Nature and Drug Repurposing. Cancer Metastasis Rev..

[21] Voutsadakis I.A., Papandreou C.N. (2012). The Ubiquitin-Proteasome System in Prostate Cancer and its Transition to Castration Resistance. Urol. Oncol..

[22] Frankland-Searby S., Bhaumik S.R. (2012). The 26S Proteasome Complex: An Attractive Target for Cancer Therapy. Biochim. Biophys. Acta.

[23] Guo Y., Cui S., Chen Y., Guo S., Chen D. (2023). Ubiquitin Specific Peptidases and Prostate Cancer. PeerJ.

[24] Lin Y., Jin X. (2024). Effect of Ubiquitin Protease System on DNA Damage Response in Prostate Cancer. Exp. Ther. Med..

[25] Hawkins P., Morton D.B., Burman O., Dennison N., Honess P., Jennings M., Lane S., Middleton V., Roughan J.V., Wells S. (2011). A Guide to Defining and Implementing Protocols for the Welfare Assessment of Laboratory Animals: Eleventh Report of the BVAAWF/FRAME/RSPCA/UFAW Joint Working Group on Refinement. Lab. Anim..

[26] Langford D.J., Bailey A.L., Chanda M.L., Clarke S.E., Drummond T.E., Echols S., Glick S., Ingrao J., Klassen-Ross T., Lacroix-Fralish M.L. (2010). Coding of Facial Expressions of Pain in the Laboratory Mouse. Nat. Methods.

[27] Sethunath D., Morusu S., Tuceryan M., Cummings O.W., Zhang H., Yin X.M., Vanderbeck S., Chalasani N., Gawrieh S. (2018). Automated Assessment of Steatosis in Murine Fatty Liver. PLoS ONE.

[28] Jordan W.H., Young J.K., Hyten M.J., Hall D.G. (2011). Preparation and Analysis of the Central Nervous System. Toxicol. Pathol..

[29] US FDA 2001 Guidance for Industry Bioanalytical Method Validation. https://www.moh.gov.bw/Publications/drug_regulation/Bioanalytical%20Method%20Validation%20FDA%202001.pdf.

[30] Bi C., Jiang B. (2018). Downregulation of RPN2 Induces Apoptosis and Inhibits Migration and Invasion in Colon Carcinoma. Oncol. Rep..

[31] Chen F., Chen X., Wang J., Zhang S., Chen M., Zhang X., Wu Z. (2022). Overexpression of SSR2 Promotes Proliferation of Liver Cancer Cells and Predicts Prognosis of Patients with Hepatocellular Carcinoma. J. Cell. Mol. Med..

[32] Cheng J., Xia L., Hao X., Gan F., Bai Y., Zhang C., Mao Y., Zhu Y., Pu Q., Park D.W. (2022). Targeting STT3A Produces an Anti-Tumor Effect in Lung Adenocarcinoma by Blocking the MAPK and PI3K/AKT Signaling Pathway. Transl. Lung Cancer Res..

[33] Liu X.-Y., Tan Q., Li L.-X. (2023). A Pan-cancer Analysis of Dyskeratosis Congenita 1 (DKC1) as A Prognostic Biomarker. Hereditas.

[34] Liu D., Luo X., Xie M., Zhang T., Chen X., Zhang B., Sun M., Wang Y., Feng Y., Ji X. (2022). HNRNPC Downregulation Inhibits IL-6/STAT3-Mediated HCC Metastasis by Decreasing HIF1A Expression. Cancer Sci..

[35] Rosenzweig R., Bronner V., Zhang D., Fushman D., Glickman M.H. (2012). Rpn1 and Rpn2 Coordinate Ubiquitin Processing Factors at Proteasome. J. Biol. Chem..

[36] Ogura M., Ayaori M., Terao Y., Hisada T., Iizuka M., Takiguchi S., Uto-Kondo H., Yakushiji E., Nakaya K., Sasaki M. (2011). Proteasomal Inhibition Promotes ATP-Binding Cassette Transporter A1 (ABCA1) and ABCG1 Expression and Cholesterol Efflux from Macrophages In Vitro and In Vivo. Arterioscler. Thromb. Vasc. Biol..

[37] Azuma Y., Takada M., Maeda M., Kioka N., Ueda K. (2009). The COP9 Signalosome Controls Ubiquitinylation of ABCA1. Biochem. Biophys. Res. Commun..

[38] Da Dalt L., Castiglioni L., Baragetti A., Audano M., Svecla M., Bonacina F., Pedretti S., Uboldi P., Benzoni P., Giannetti F. (2021). PCSK9 Deficiency Rewires Heart Metabolism and Drives Heart Failure with Preserved Ejection Fraction. Eur. Heart J..

[39] Otto G.P., Rathkolb B., Oestereicher M.A., Lengger C.J., Moerth C., Micklich K., Fuchs H., Gailus-Durner V., Wolf E., Hrabě de Angelis M. (2016). Clinical Chemistry Reference Intervals for C57BL/6J, C57BL/6N, and C3HeB/FeJ Mice (*Mus. musculus*). J. Am. Assoc. Lab. Anim. Sci..

[40] Grander C., Grabherr F., Tilg H. (2023). Non-alcoholic Fatty Liver Disease: Pathophysiological Concepts and Treatment Options. Cardiovasc. Res..

[41] Jakielska E., Głuszak P., Walczak M., Bryl W. (2023). Effects of PCSK9 Inhibitors on Metabolic-associated Fatty Liver Disease: A Short Review. Prz. Gastroenterol..

[42] Olson H., Betton G., Robinson D., Thomas K., Monro A., Kolaja G., Lilly P., Sanders J., Sipes G., Bracken W. (2000). Concordance of the Toxicity of Pharmaceuticals in Humans and in Animals. Regul. Toxicol. Pharmacol..

[43] Silva-Santana G., Bax J.C., Fernandes D.C.S., Bacellar D.T.L., Hooper C., Dias A., Silva C.B., de Souza A.M., Ramos S., Santos R.A. (2020). Clinical Hematological and Biochemical Parameters in Swiss, BALB/c, C57BL/6 and B6D2F1 Mus Musculus. Anim. Model. Exp. Med..

[44] He Y.-Q., Wei Y.-Q., Huang G.-M., Liu G.-P., Lin Z.-Q., Liu T.-T., Jiang X., Lu J.-J. (2024). Sex Differences in LDL-C Reduction Response to Evolocumab: A Propensity Score Matching Analysis. Pharmacotherapy.

[45] Chapman M.E., Hu L., Plato C.F., Kohan D.E. (2010). Bioimpedance Spectroscopy for the Estimation of Body Fluid Volumes in Mice. Am. J. Physiol. Renal Physiol..

[46] Kim Y.-S., Yi B.-R., Kim N.-H., Choi K.-C. (2014). Role of the Epithelial–Mesenchymal Transition and Its Effects on Embryonic Stem Cells. Exp. Mol. Med..

[47] Jiang L., Liu X., Kolokythas A., Yu J., Wang A., Heidbreder C.E., Shi F., Zhou X. (2010). Downregulation of the Rho GTPase Signaling Pathway is Involved in the microRNA-138-Mediated Inhibition of Cell Migration and Invasion in Tongue Squamous Cell Carcinoma. Int. J. Cancer.

[48] Aliabadi F., Sohrabi B., Mostafavi E., Pazoki-Toroudi H., Webster T.J. (2021). Ubiquitin-Proteasome System and the Role of its Inhibitors in Cancer Therapy. Open Biol..

[49] Liu C., Lou W., Yang J.C., Liu L., Armstrong C.M., Lombard A.P., Zhao R., Noel O.D.V., Tepper C.G., Chen H.-W. (2018). Proteostasis by STUB1/HSP70 Complex Controls Sensitivity to Androgen Receptor Targeted Therapy in Advanced Prostate Cancer. Nat. Commun..

[50] AVMA Guidelines on Euthanasia 2020. https://www.avma.org/sites/default/files/2020-02/Guidelines-on-Euthanasia-2020.pdf.

[51] OECD (2018). Test No. 408: Repeated Dose 90-Day Oral Toxicity Study in Rodents.

[52] Zhang Y., Huo M., Zhou J., Xie S. (2010). PKSolver: An Add-In Program for Pharmacokinetic and Pharmacodynamic Data Analysis in Microsoft Excel. Comput. Methods Programs Biomed..

[53] Andrews S. FastQC: A Quality Control Tool for High Throughput Sequence Data.

[54] Dobin A., Davis C.A., Schlesinger F., Drenkow J., Zaleski C., Jha S., Batut P., Chaisson M., Gingeras T.R. (2013). STAR: Ultrafast Universal RNA-seq Aligner. Bioinformatics.

[55] Liao Y., Smyth G.K., Shi W. (2014). FeatureCounts: An Efficient General-Purpose Program for Assigning Sequence Reads to Genomic Features. Bioinformatics.

[56] Love M.I., Huber W., Anders S. (2014). Moderated Estimation of Fold Change and Dispersion for RNA-seq Data with DESeq2. Genome Biol..

[57] Korthauer K., Kimes P.K., Duvallet C., Reyes A., Subramanian A., Teng M., Shukla C., Alm E.J., Hicks S.C. (2019). A Practical Guide to Methods Controlling False Discoveries in Computational Biology. Genome Biol..

[58] Subramanian A., Tamayo P., Mootha V.K., Mukherjee S., Ebert B.L., Gillette M.A., Paulovich A., Pomeroy S.L., Golub T.R., Lander E.S. (2005). Gene Set Enrichment Analysis: A Knowledge-Based Approach for Interpreting Genome-Wide Expression Profiles. Proc. Natl. Acad. Sci. USA.

[59] Ashburner M., Ball C.A., Blake J.A., Botstein D., Butler H., Cherry J.M., Davis A.P., Dolinski K., Dwight S.S., Eppig J.T. (2000). Gene Ontology: Tool for the Unification of Biology. Nat. Genet..

[60] Aleksander S.A., Balhoff J., Carbon S., Cherry J.M., Drabkin H.J., Ebert D., Feuermann M., Gaudet P., Harris N.L., Hill D.P. (2023). The Gene Ontology Knowledgebase in 2023. Genetics.

[61] Kanehisa M., Furumichi M., Sato Y., Kawashima M., Ishiguro-Watanabe M. (2023). KEGG for Taxonomy-Based Analysis of Pathways and Genomes. Nucleic Acids Res..

[62] Yu G. (2024). Thirteen Years of ClusterProfiler. Innovation.

[63] Xu S., Hu E., Cai Y., Xie Z., Luo X., Zhan L., Tang W., Wang Q., Liu B., Wang R. (2024). Using ClusterProfiler to Characterize Multiomics Data. Nat. Protoc..

[64] Wu T., Hu E., Xu S., Chen M., Guo P., Dai Z., Feng T., Zhou L., Tang W., Zhan L. (2021). ClusterProfiler 4.0: A Universal Enrichment Tool for Interpreting Omics Data. Innovation.

[65] EnrichR. http://cran.r-project.org/package=enrichR.

[66] Yu G., Wang L.G., Han Y., He Q.Y. (2012). ClusterProfiler: An R Package for Comparing Biological Themes Among Gene Clusters. Omics.

[67] Yu G., Wang L.G., Yan G.R., He Q.Y. (2015). DOSE: An R/Bioconductor Package for Disease Ontology Semantic and Enrichment Analysis. Bioinformatics.

[68] Wickham H., Averick M., Bryan J., Chang W., McGowan L.D., François R., Grolemund G., Hayes A., Henry L., Hester J. (2019). Welcome to the Tidyverse. J. Open Source Softw..

[69] Doncheva N.T., Morris J.H., Gorodkin J., Jensen L.J. (2019). Cytoscape StringApp: Network Analysis and Visualization of Proteomics Data. J. Proteome Res..

[70] Shannon P., Markiel A., Ozier O., Baliga N.S., Wang J.T., Ramage D., Amin N., Schwikowski B., Ideker T. (2003). Cytoscape: A Software Environment for Integrated Models of Biomolecular Interaction Networks. Genome Res..

