# Supplementary material for: Safety, Pharmacokinetics, Translational and Molecular Mechanistic Insights on the Prostate Cancer Recurrence Suppressor Pseurotin A
Source: Molecules. 2025 Oct 2;30(19):3963. doi: 10.3390/molecules30193963 (PMC12525728; doi:10.3390/molecules30193963)
Supplement: Supplementary file 1 [file molecules-30-03963-s001.zip › molecules-3825260-supplementary.pdf]

molecules

Supplementary Material

Safety, Pharmacokinetics, Translational and Molecular Mechanistic Insights on The Prostate Cancer Recurrence Suppressor Pseurotin A

Oliver C. McGehee [1], Hassan Y. Ebrahim [1], Sharon Meyer [1,†], Nehal A. Ahmed [1], Chandra Mohan Reddy Muthumula [1], Dalal Dawud [1], Judy A. King [2], Amal Kaddoumi [3], Khalid A. El Sayed [1,*]

1 Department of Basic Pharmaceutical and Toxicological Sciences, College of Pharmacy, University of Louisiana at Monroe, 1800 Bienville Drive, Monroe, LA 71201, USA; mcgeheoc@warhawks.ulm.edu (O.C.M); hebrahim@vcom.edu (H.Y.E.); meyer@ulm.edu (S.M.); atefkhaledahmedabdn@warhawks.ulm.edu (N.A.A.); mcmohanreddy8@gmail.com (C.M.R.M.); dawuddr@warhawks.ulm.edu (D.D.).
2 Department of Biomedical Sciences, Discipline of Pharmacology, Edward Via College of Osteopathic Medicine, Monroe, LA, USA.
3 DeBusk College of Osteopathic Medicine, Lincoln Memorial University, 9737 Cogdill Road, Knoxville, TN 37932, USA; judy.king@lmunet.edu (J.A.K.)
4 Department of Pharmacology and Toxicology, Medical College of Georgia, Augusta University, 1201 Goss Lane, Augusta, GA 30912, USA; akaddoumi@augusta.edu (A.K.).
† Deceased on July 24th, 2023.

* Correspondence: elsayed@ulm.edu (K.A.E.); Tel.: +1-318-342-1725. orcid.org/0000-0002-1456-4064.

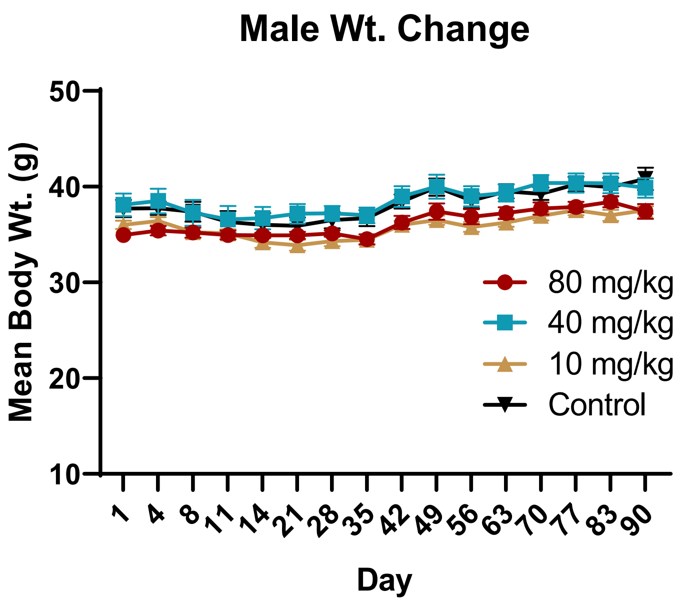

Supplementary Figure S1. The 90-day daily oral dose PsA (mean ± SEM) treatment effects on body weight of male *Swiss albino* mice.

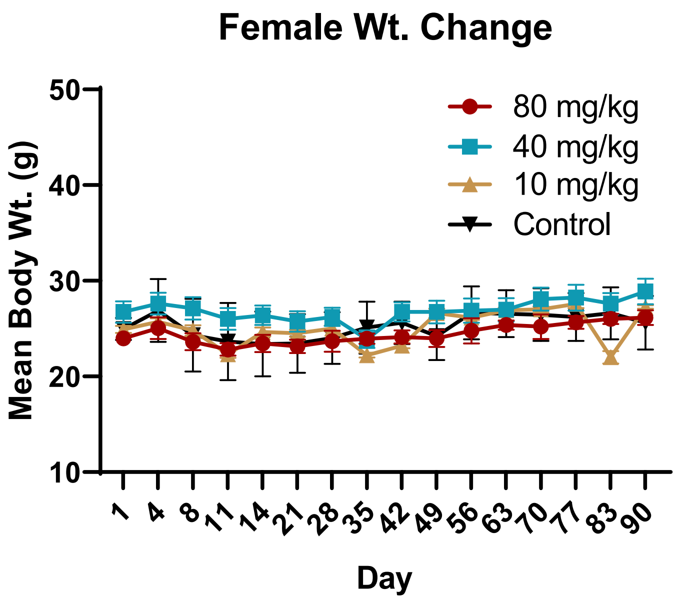

Supplementary Figure S2. The 90-day daily oral dose PsA (mean ± SEM) treatment effects on body weight of female *Swiss albino* mice.

Supplementary Table S1. Relative organ weight (mean ± SD) of male *Swiss albino* mice in PsA chronic toxicity study.

Parameters	Control			10mg/kg			40mg/kg			80mg/kg		
Liver	1883	±	273.7	1881	±	134.3	1869	±	295.8	1946	±	272.4
Kidneys	627.2	±	80.2	684.7	±	41.0	747.5	±	92.3	673.7	±	50.1
Spleen	100.5	±	18.7	114.7	±	21.4	118.5	±	3.9	108.7	±	12.1
Lungs	295.2	±	52.6	288.8	±	62.4	302.1	±	23.5	209.9	±	61.0
Heart	404.7	±	59.4	248.1	±	54.6	261.7	±	25.2	320.8	±	58.9
Brain	394.4	±	37.9	394.4	±	37.9	413.6	±	54.5	409.2	±	28.3

Supplementary Table S2. Relative organ weight (mean ± SD) of female *Swiss albino* mice in PsA chronic toxicity study.

Parameters	Control			10mg/kg			40mg/kg			80mg/kg		
Liver	1375	±	163.3	1218	±	31.6	1311	±	135.7	1369	±	146.3
Kidneys	447.3	±	38.9	475.9	±	31.8	470.4	±	87.8	461.9	±	20.6
Spleen	124.3	±	48.1	106.3	±	48.4	125.1	±	22.6	109.7	±	20.2
Lungs	215.5	±	54.2	211.7	±	40.2	229.0	±	37.1	236.9	±	66.2
Heart	229.3	±	39.4	197.7	±	33.5	186.2	±	12.3	231.1	±	24.4
Brain	397.7	±	52.8	438.5	±	18.9	355.1	±	25.2	376.7	±	47.4

Supplementary Table S3. Biochemical parameters glucose (GLU), aspartate aminotransferase (AST), alanine aminotransferase (ALT), alkaline phosphatase (ALP), blood urea nitrogen (BUN), and creatinine (CREAT) (mean ± SEM) of male *Swiss albino* mice in chronic toxicity study of PsA.

Parameters	Control			10mg/kg			40mg/kg			80mg/kg		
**GLU (mg/dL)**	248.5	±	15.7	263.8	±	9.9	248.5	±	12.0	272.3	±	15.4
**AST (U/L)**	150.8	±	43.5	57.8	±	12.8	42.8	±	1.9	48.5	±	4.50
**ALT (U/L)**	40.8	±	4.0	28.3	±	1.7	28.5	±	2.5	48.0	±	4.8
**ALP (U/L)**	84.3	±	15.5	71.8	±	4.7	78.3	±	5.2	63.0	±	3.4
**BUN (mg/dL)**	22.0	±	0.7	18.3	±	0.7	20.5	±	0.8	20.3	±	0.2
**CREAT (mg/dL)**	<0.2	±	0.0	0.2	±	0.0	0.2	±	0.0	0.2	±	0.0

Supplementary Table S4. Biochemical parameters glucose (GLU), aspartate aminotransferase (AST), alanine aminotransferase (ALT), alkaline phosphatase (ALP), blood urea nitrogen (BUN), and creatinine (CREAT) (mean ± SEM) of female *Swiss albino* mice in chronic toxicity study of PsA.

Parameters	Control			10mg/kg			40mg/kg			80mg/kg		
**GLU (mg/dL)**	228.3	±	15.8	291.7	±	6.5	259.0	±	5.7	235.3	±	19.4
**AST (U/L)**	197.3	±	33.6	61.7	±	6.4	67.8	±	7.1	81.3	±	17.8
**ALT (U/L)**	48.3	±	5.8	34.3	±	1.9	34.5	±	1.9	30.3	±	3.4
**ALP (U/L)**	77.7	±	12.1	78.0	±	3.8	68.0	±	3.8	86.8	±	13.4
**BUN (mg/dL)**	24.3	±	2.5	22.0	±	0.6	17.5	±	0.4	18.5	±	2.1
**CREAT (mg/dL)**	<0.2	±	0.0	0.2	±	0.0	<0.2	±	0.0	0.2	±	0.0

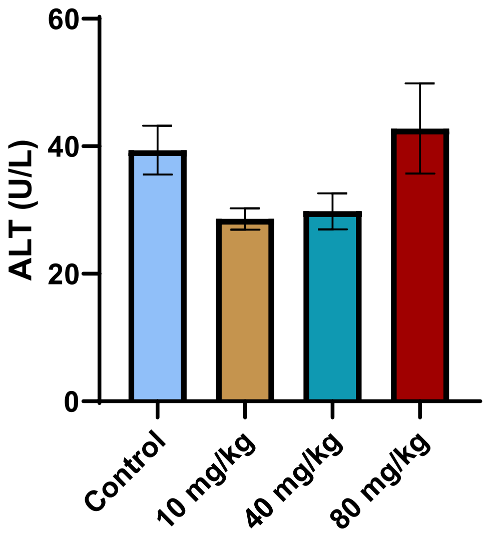

Supplementary Figure S3. Effects of PsA treatments on plasma ALT levels (mean ± SEM) in male *Swiss albino* mice

Supplementary Table S5. Hematological parameters (mean ± SD) of male *Swiss albino* mice in chronic toxicity study of PsA.

Parameters	Control	10mg/kg	40mg/kg	80mg/kg
**WBC-H (10^3/μL)**	6.7 ± 1.4	4.5 ± 1.0	5.0 ± 1.8	7.9 ± 1.4
**RBC-H (10^6/μL)**	8.6 ± 0.5	8.3 ± 0.6	8.6 ± 0.8	9.0 ± 0.1
**HGB (g/dL)**	12.2 ± 0.7	11.8 ± 0.4	11.9 ± 0.9	12.5 ± 0.3
**HCT (%)**	41.5 ± 4.3	41.0 ± 3.0	47.4 ± 4.3	47.8 ± 4.9
**MCV (fL)**	48.2 ± 2.2	49.6 ± 2.5	55.4 ± 4.8	53.3 ± 5.1
**MCHC (g/dL)**	29.5 ± 4.3	28.8 ± 2.5	25.1 ± 3.3	26.3 ± 2.2
**PLT (10^3/μL)**	477.0 ± 125.6	555.0 ± 147.7	413.0 ± 111.2	580.3 ± 119.8

Supplementary Table S6. Hematological parameters (mean ± SD) of female *Swiss albino* mice in chronic toxicity study of PsA.

Parameters	Control	10mg/kg
**WBC-H (10^3/μL)**	7.1 ± 1.6	6.5 ± 0.6
**RBC-H (10^6/μL)**	10.2 ± 0.4	9.3 ± 0.3
**HGB (g/dL)**	13.4 ± 0.5	12.5 ± 0.3
**HCT (%)**	56.1 ± 4.2	48.5 ± 6.0
**MCV (fL)**	54.9 ± 5.3	52.1 ± 5.0
**MCHC (g/dL)**	24.0 ± 2.4	26.2 ± 3.0
**PLT (10^3/μL)**	477.3 ± 73.8	459.0 ± 99.1

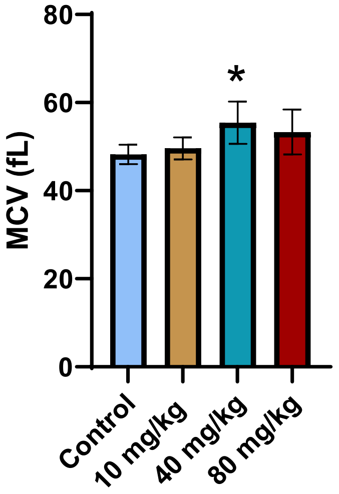

Supplementary Figure S4. Effects of PsA treatments on mean corpuscular volume in male *Swiss albino* mice. Samples were collected in K2 EDTA blood collection tubes to prevent clotting. Hematological parameters were determined using the Siemens Advia 120 hematology analyzer. Results for the hematological parameters were analyzed separately by One-way Analysis of Variance (ANOVA) followed by multiple comparison with Dunnett's test * indicates a statistical significance at $p < 0.05$.

.

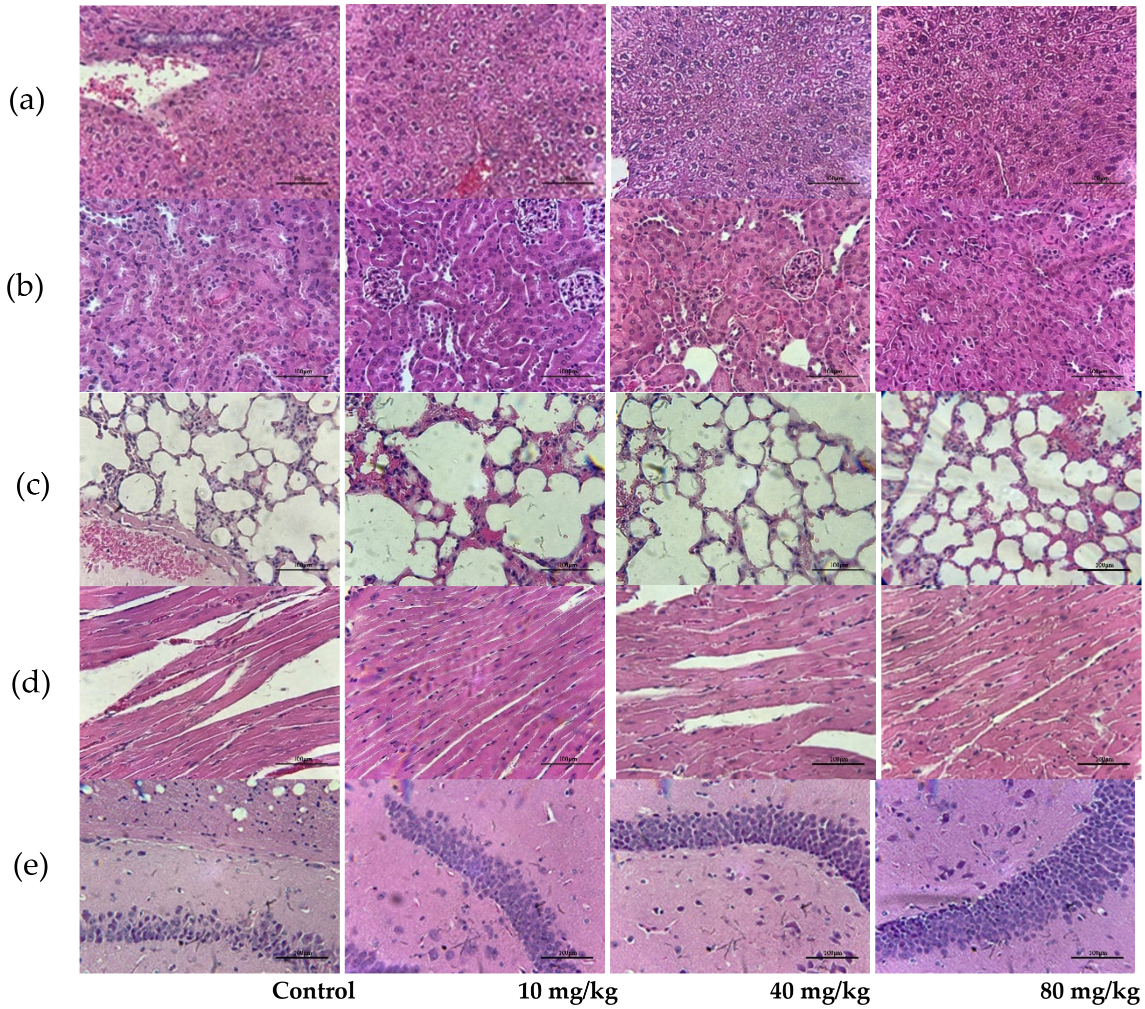

Supplementary Figure S5. Histopathological examination of *Swiss albino* female mice treated with differing

concentrations of PsA in 90-day oral dose chronic toxicity study. (**a**) H&E stained liver sections taken at 400x magnification. The bile ducts, hepatic arteries, and portal veins remained structurally intact. Kupffer cells were found lining the sinusoids and did not show overexpression in the treatment groups, and early signs of inflammation were not seen in any of the liver sections. (**b**) H&E stained renal cortex sections taken at 400x magnification. In all treatment groups, the glomerulus was structurally homogeneous with a clearly visible bowman's capsule. The proximal tubules contained no observable desquamation or signs of necrosis, and the collecting ducts and loops of Henle retained structural integrity. (**c**) H&E stained bronchiolar sections taken at 400x magnification. For both the control and treatment groups, the alveolar sacs displayed no structural alterations and were free of cellular debris or red blood cell migration. The epithelium of the alveolar ducts displayed a clear nucleus with no sloughing off of tissue. (**d**) H&E stained cardiac muscle sections taken at 400x magnification. The intercalated discs, intercellular junctions that connect adjacent cardiac muscle cells, were intact and clearly visible and the large oval nuclei of the cardiomyocytes were healthy and centrally located (**e**) H&E stained hippocampal sections at 400x magnification. The pyramidal neuronal layer of the mouse hippocampus was uniformly arranged and structurally intact, and healthy glial cells were present in the molecular layer. (Scale bar: 100 μm).

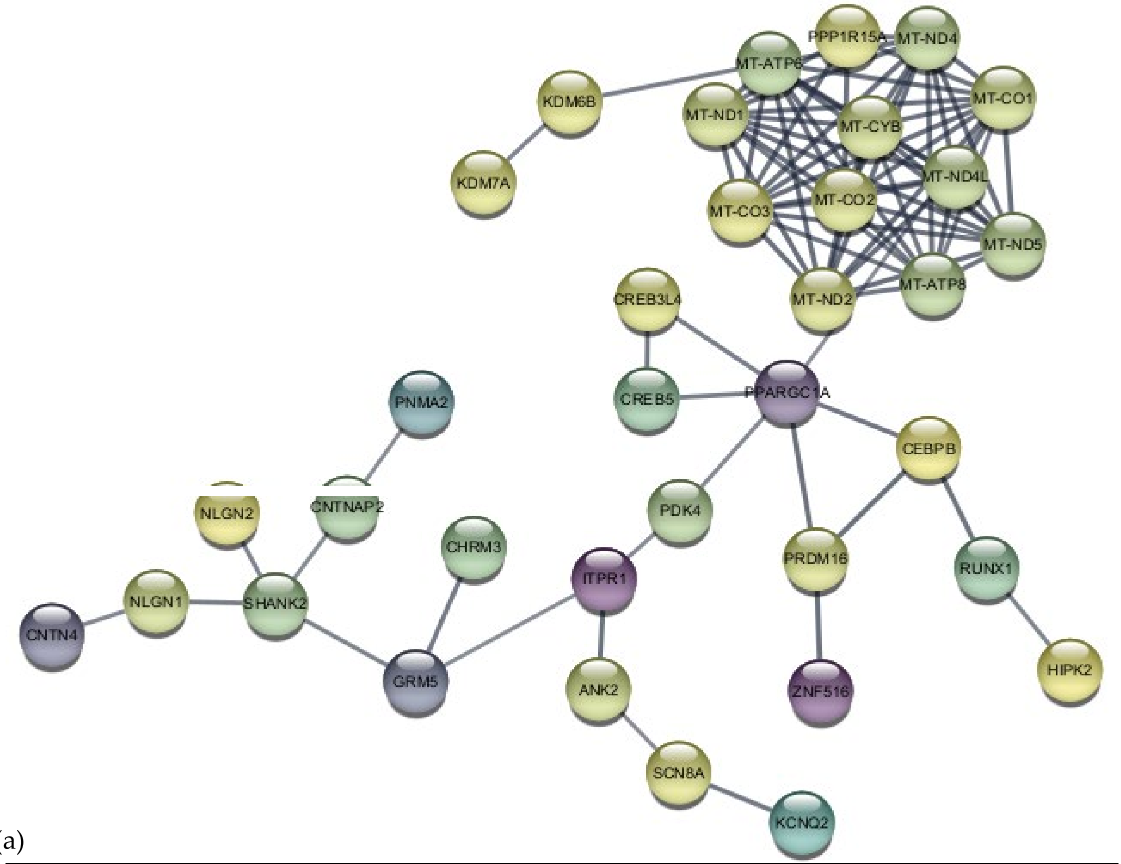

(a)

(b)

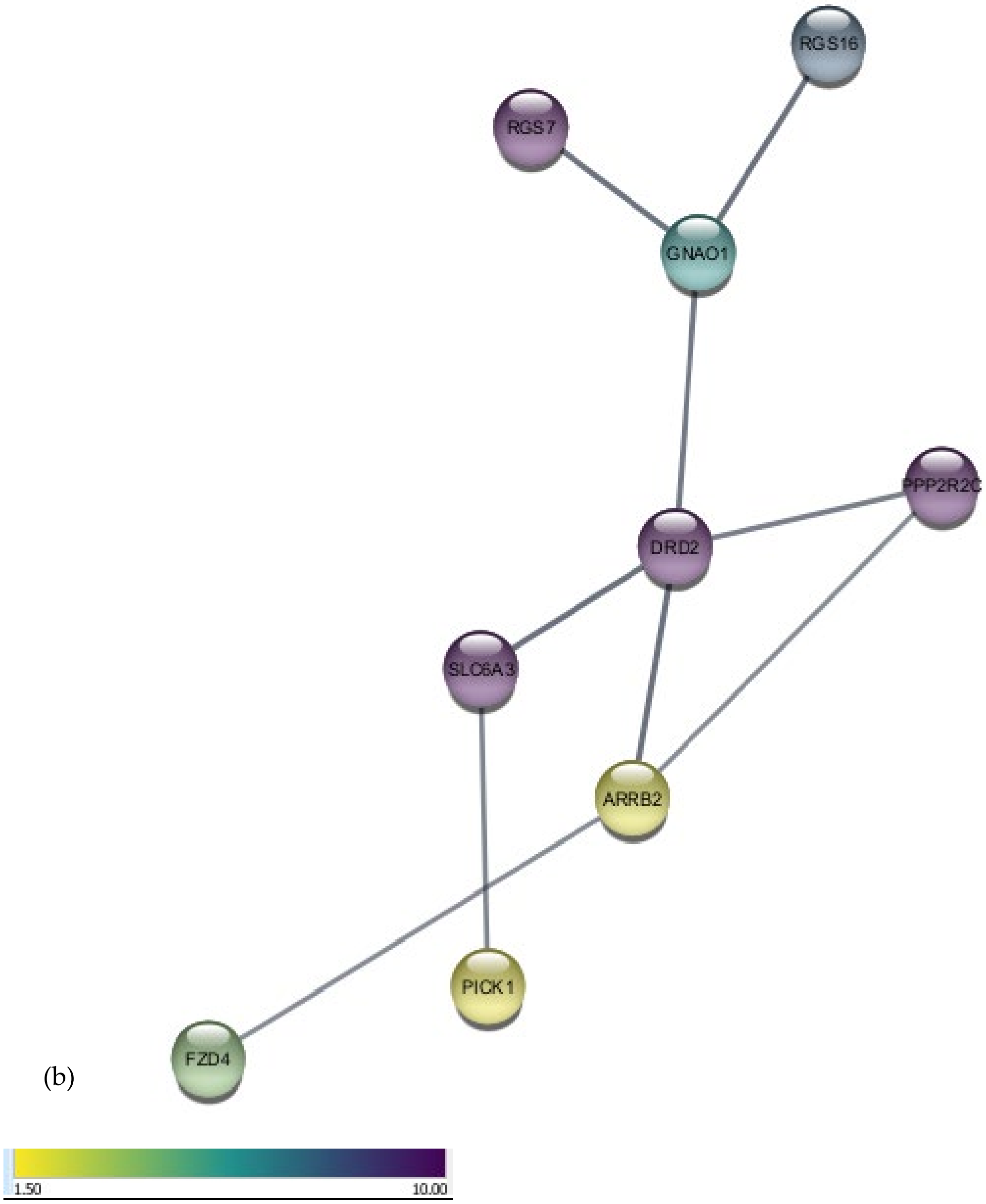

Supplementary Figure S6. STRING functional network analysis of upregulated DEGs comparing the PsA versus VC-treated CWR-R1ca tumors cells. The cutoff criteria for the DEGs were log2FC ≥1.5 and log2FC ≤-1.5, adjusted *p*-value ≤0.05, and FDR ≤0.1. The cutoff criteria for the STRING functional network analysis was 0.8. (**a**) The most prominent network displaying 35 nodes and 83 edges. (**b**) Second most prominent network representing 9 nodes and 9 edges. Circular nodes were colored using a continuous mapping color gradient based on Log2FC ranging from 1.5 to 10 or greater.
